# The integrative epigenomic-transcriptomic landscape of ER positive breast cancer

**DOI:** 10.1186/s13148-015-0159-0

**Published:** 2015-12-09

**Authors:** Yang Gao, Allison Jones, Peter A. Fasching, Matthias Ruebner, Matthias W. Beckmann, Martin Widschwendter, Andrew E. Teschendorff

**Affiliations:** CAS Key Lab for Computational Biology, CAS-MPG Partner Institute for Computational Biology, Chinese Academy of Sciences, Shanghai Institute for Biological Sciences, 320 Yue Yang Road, 200031 Shanghai, China; Department of Women’s Cancer, University College London, 74 Huntley Street, London, WC1E 6BT UK; University Clinic Erlangen, Department of Gynaecology and Obstetrics, Friedrich-Alexander University Erlangen-Nuremberg, Erlangen, 91054 Germany; Statistical Genomics Group, Paul O’Gorman Building, UCL Cancer Institute, University College London, 72 Huntley Street, London, WC1E 6BT UK

## Abstract

**Background:**

While recent integrative analyses of copy number and gene expression data in breast cancer have revealed a complex molecular landscape with multiple subtypes and many oncogenic/tumour suppressor driver events, much less is known about the role of DNA methylation in shaping breast cancer taxonomy and defining driver events.

**Results:**

Here, we applied a powerful integrative network algorithm to matched DNA methylation and RNA-Seq data for 724 estrogen receptor (ER)-positive (ER+) breast cancers and 111 normal adjacent tissue specimens from The Cancer Genome Atlas (TCGA) project, in order to identify putative epigenetic driver events and to explore the resulting molecular taxonomy. This revealed the existence of nine functionally deregulated epigenetic hotspots encompassing a total of 146 genes, which we were able to validate in independent data sets encompassing over 1000 ER+ breast cancers. Integrative clustering of the matched messenger RNA (mRNA) and DNA methylation data over these genes resulted in only two clusters, which correlated very strongly with the luminal-A and luminal B subtypes. Overall, luminal-A and luminal-B breast cancers shared the same epigenetically deregulated hotspots but with luminal-B cancers exhibiting increased aberrant DNA methylation patterns relative to normal tissue. We show that increased levels of DNA methylation and mRNA expression deviation from the normal state define a marker of poor prognosis. Our data further implicates epigenetic silencing of WNT signalling antagonists and bone morphogenetic proteins (BMP) as key events underlying both luminal subtypes but specially of luminal-B breast cancer. Finally, we show that DNA methylation changes within the identified epigenetic interactome hotspots do not exhibit mutually exclusive patterns within the same cancer sample, instead exhibiting coordinated changes within the sample.

**Conclusions:**

Our results indicate that the integrative DNA methylation and transcriptomic landscape of ER+ breast cancer is surprisingly homogeneous, defining two main subtypes which strongly correlate with luminal-A/B subtype status. In particular, we identify WNT and BMP signalling as key epigenetically deregulated tumour suppressor pathways in luminal ER+ breast cancer, with increased deregulation seen in luminal-B breast cancer.

**Electronic supplementary material:**

The online version of this article (doi:10.1186/s13148-015-0159-0) contains supplementary material, which is available to authorized users.

## Background

Large-scale integrative analyses of copy number and gene expression data in breast cancer have revealed a complex molecular landscape with many putative driver events [1–4]. The most prominent driver events identified (or confirmed) by these studies include amplification of oncogenes like *ERBB2*, *CCND1*, *ZNF218* or *EMSY*, as well as deletion of tumour suppressors like *TP53* and *PTEN*. The largest study to date, performed by the Molecular Taxonomy of Breast Cancer International Consortium (METABRIC), suggests that many more putative driver events exist, with many of these being specific to certain breast cancer subtypes [4]. By clustering over all copy number-driven gene expression changes, this same study further exposed the underlying complexity, inferring the existence of at least 10 breast cancer subtypes, called intrinsic clusters (IC) [4, 5].

Another mechanism that can lead to deregulation of gene expression in cancer is aberrant DNA methylation. In particular, changes in DNA methylation patterns occurring in the vicinity of gene promoters have long been recognized as a mechanism of tumour suppressor inactivation or oncogene activation [6–8]. In spite of this, the integrative analysis of DNA methylation and gene expression data in breast cancer remains largely unexplored [2, 9]. In fact, to date, the largest study to perform DNA methylation profiling of breast cancer revealed five methylation subtypes with three of these clusters correlating with three of the well-known intrinsic subtypes of breast cancer, specifically, with the basal, luminal-A and luminal-B subtypes [2]. However, relatively little is known about how breast cancers would cluster if we were to perform an explicit integrative analysis of DNA methylation and gene expression.

Thus, we here aimed to perform a comprehensive analysis of the integrative DNA methylation gene expression landscape of breast cancer. In doing so, we wanted to address the following key questions. First, how does the molecular taxonomy of breast cancer look like when viewed from the perspective of DNA methylation-driven gene expression changes? Second, are their specific signalling pathways which are epigenetically deregulated and how does this epigenetic deregulation vary across breast cancer subtypes? Third, do putative epigenetic driver events targeting specific gene modules or signalling pathways exhibit a mutually exclusive pattern of deregulation within individual tumours, similar to what is observed at the copy number level [10]? In order to address these questions, we decided to apply a functional supervised algorithm, called Functional Epigenetic Modules (FEMs) [11, 12], which performs an integrative analysis of DNA methylation and gene expression data at a system level. The system-level integration is done using a comprehensive high-quality protein-protein interactome (PPI) as a scaffold, whereby functionally related genes occupy proximal positions in the network. The FEM algorithm then infers interactome hotspots of simultaneous differential DNA methylation and expression, allowing putative driver events to be identified [11].

The feasibility of the FEM algorithm to uncover driver events in cancer was demonstrated by us previously [11, 12]. Indeed, perhaps one of the clearest examples of an epigenetic aberration constituting a driver event was found in the context of endometrial cancer [12]. Specifically, this study showed that DNA methylation-induced silencing of a gene called *HAND2* was causally implicated in endometrial carcinogenesis. In fact, it was shown how DNA methylation-induced suppression of *HAND2* inactivates the progesterone receptor signalling pathway, which is the key tumour suppressor pathway in this type of cancer. Integration of DNA methylation data with a human interactome was also shown to be a fruitful approach leading to novel insights in other contexts. For instance, DNA methylation changes occurring in ageing do not happen randomly in the context of protein interaction networks [13], but tend to cluster in such networks, targeting specific pathways, in the same way as gene expression changes do in the context of disease phenotypes [14]. Given the success of FEM to identify causal epigenetic events in endometrial cancer and key pathways in ageing, we were impelled to explore the systematic application of FEM in the context of breast cancer and specifically to estrogen receptor (ER)-positive (ER+) breast cancer.

## Results

### Identification and validation of FEM modules in ER+ breast cancer

**Fig. 1:**
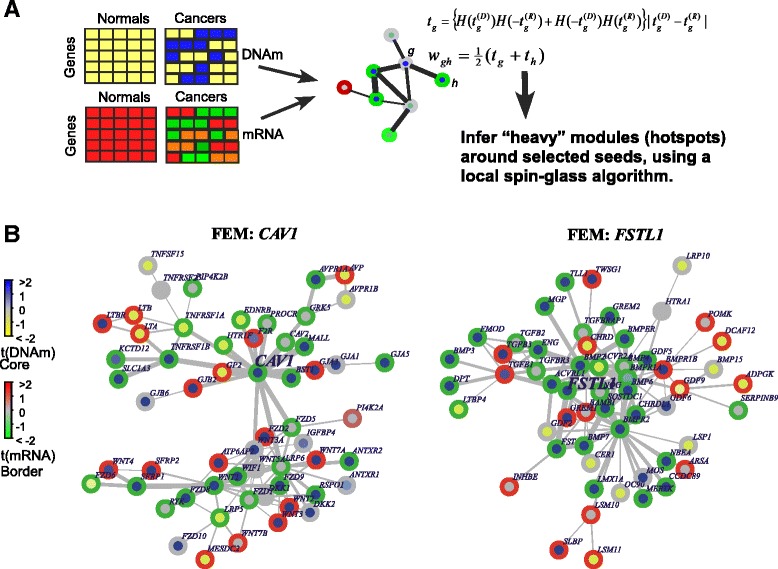
The FEM algorithm and examples of FEM modules in ER+ breast cancer. **a** The FEM algorithm first uses gene-centred statistics of differential DNA methylation, *t*_*g*_^(*D*)^ and differential mRNA expression, *t*_*g*_^(*R*)^, here between normal and ER+ breast cancer, to weight the edges in a PPI network. The weight of the edge between gene *g* and *h* is constructed as indicated, where *H*(*x*) denotes the Heaviside function (*H*(*x*) = 1 if *x* > 0, *H*(*x*) = 0 if *x* < 0), which are being used to impose an anti-correlation. Hotspots of differential DNAm and mRNA expression are then inferred by running a module detection algorithm on the PPI network, which attempts to find subnetworks that maximize the modularity (average weight density) locally. **b** Examples of two FEMs centred around seed genes *CAV1* and *FSTL1* in ER+ breast cancer

Given that the process of cellular differentiation is dictated by the specific activation/deactivation of signalling pathways and that this is largely controlled by epigenetics, we reasoned that putative epigenetic drivers of cancer could be identified by integrating DNA methylation with gene expression data in the context of a functional gene network, which incorporates pathway-level information, such as that provided by a comprehensive high-quality human protein interactome [15]. In order to identify interactome hotspots of simultaneous differential methylation and gene expression associated with ER+ breast cancer, we applied our FEM algorithm [11] (Fig. 1a) to the ER+ breast cancer subset of The Cancer Genome Atlas (TCGA), encompassing Illumina Infinium 450K DNA methylation and RNA-Seq data for 111 normal adjacent and 724 cancer samples. After removing largely overlapping (and therefore redundant) modules, this resulted in the identification of nine non-redundant FEM modules, consisting of 257 unique genes (Table 1, Methods, Additional file 1: Tables S1–S2). Of these 257 genes, 146 were significantly differentially methylated and expressed, with 99 of these (i.e. 68 %) exhibiting an anti-correlation between DNA methylation and gene expression. Thus, the FEMs were driven on average by 38 % (99/257) of the genes making up the modules. Network representations of the FEMs confirmed that they contained a significant amount of potential epigenetic deregulation (Fig. 1b, Additional file 1: Figure S1). We verified that the proportions of differentially methylated and differential expressed genes in each FEM were not strongly biased to one particular data type, thus confirming that FEM is effective in avoiding such bias (Additional file 1: Figure S2).

**Table 1:** FEMs associated with ER+ breast cancer as inferred in the TCGA data set

FEM seed	Size	Mod (TCGA)	*P* (TCGA)	Mod (VAL)	*P* (VAL)
TGFB1I1	13	4.94	0.018	3.32	0.013
KRT18	47	4.37	0.001	1.9	0.191
LEP	13	4.57	0.049	2.89	0.045
CCL11	12	5.13	0.018	3.23	0.018
FAM107A	16	4.57	0.036	1.71	0.36
PROC	18	7.06	<0.001	1.68	0.374
FSTL1	56	3.91	0.033	2.00	0.125
MME	26	4.97	0.003	2.13	0.122
CAV1	58	3.65	0.046	2.83	0.003

Columns label the seed gene symbol of the FEM, the size of FEM, its modularity (Mod) and *P* value (*P*) in the TCGA set, as well as the modularity and *P* values in the validation (VAL) set

**Fig. 2:**
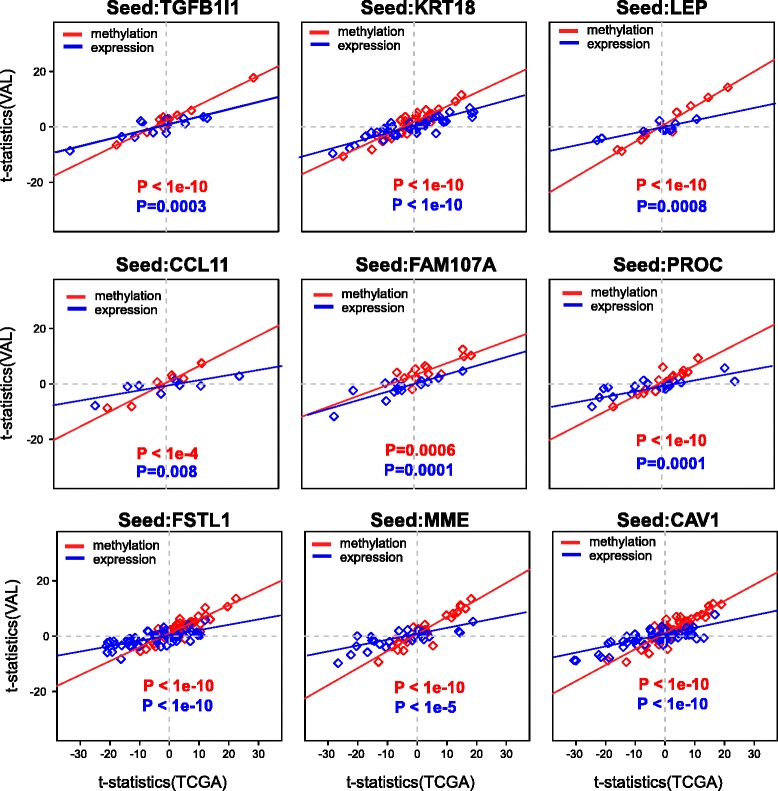
Validation of FEM module gene *t*-statistics. For each FEM module, a scatterplot of *t*-statistics of differential DNAm (*red*) and mRNA expression (*blue*) in discovery TCGA set (*x*-axis) against the corresponding statistics in the validation set (*y*-axis). Linear regression lines and *P* values are given

In order to validate these FEMs, we collected independent data encompassing Illumina 450K DNA methylation profiles for 49 normal breast and 254 ER+ samples, as well as gene expression for 13 normal breast and 110 ER+ samples (Methods). Validation was performed in two different ways to assess both the hotspot nature of the FEM modules in the independent data and also to assess the consistency of the differential methylation and expression statistics across studies. Of the nine FEMs, four exhibited significant modularity scores in the independent data (Table 1), thus confirming their hotspot nature. Although the other five FEMs did not pass significance, their *P* values were always smaller than 0.5, indicating that their non-significance could be due to other factors such as the unmatched nature of the independent DNA methylation (DNAm) and messenger RNA (mRNA) data. Indeed, scatterplots of *t*-statistics of differential methylation (and differential expression) between the TCGA and validation sets revealed strong agreement at both DNAm and mRNA levels for all FEM modules (Fig. 2).

In order to shed light on the biological significance of the FEM modules, we performed GSEA using the Molecular Signatures Database (MSigDB) [16]. The top two enriched pathways mapped to the WNT and TGF-beta signalling pathways (Table 2). In particular, we identified 22 genes within the CAV1 FEM, which all mapped to the WNT signalling pathway, with 10 of these significant at either DNAm or mRNA levels (*P* < 0.05) and with 5 exhibiting simultaneous hypermethylation and underexpression (Fig. 1b). The FSTL1 FEM contained 18 genes mapping to the TGF-beta signalling pathway, with 7 of these exhibiting simultaneous hypermethylation and underexpression (Fig. 1b).

**Table 2:** GSEA on FEM modules revealed strong enrichment of two FEMs with seed genes CAV1 and FSTL1, respectively

FEM	Size	Enriched pathway	Overlap	Overlapping genes
CAV1	58	WNT signalling	23	DKK1 WNT3A FZD9 WNT2 WNT7A IGFBP4
				FZD8 FZD1 LRP6 FZD2 LRP5 FZD6 RSPO1
				WNT5A RYK FZD5 ATP6AP2 WNT3 WIF1
				WNT1 WNT7B FZD10 SFRP1
FSTL1	56	TGF-beta signalling	18	NOG GDF5 CHRD TGFB2 TGFB1 FST TGFB3
				BMPR2 GDF6 BMPR1A BMPR1B ACVRL1
				ACVR2A BMP4 BMP2 BMP7 BMP6 INHBE

Columns label the FEM module size, the main enriched pathway, the number of overlapping genes between FEM and pathway and the overlapping genes

### Integrative clustering correlates strongly with luminal subtypes

**Fig. 3:**
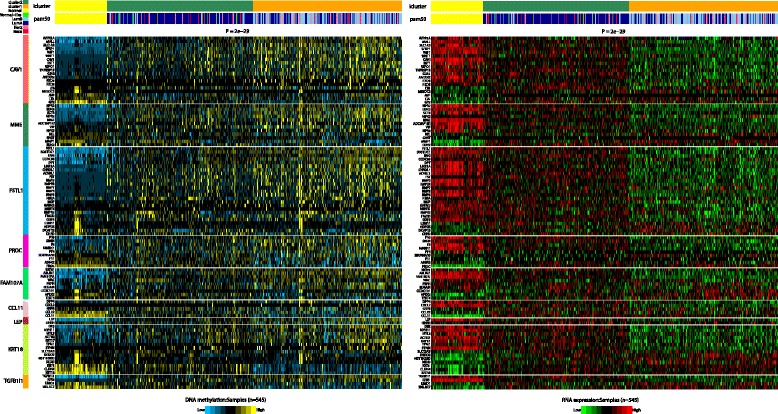
Integrative clustering of matched DNAm and mRNA data in ER+ breast cancer. Result of the integrative iCluster algorithm on 545 ER+ breast cancer samples with matched DNAm and mRNA expression. The algorithm predicted two main clusters, which are depicted together with the normal samples, as indicated in the *top barcode*. The *second barcode* shows the distribution of PAM50 intrinsic subtypes across the clusters. Genes have been ordered according to the FEM module they belong too, as indicated. *Left heatmap* is for DNAm, *right heatmap* for mRNA expression

Next, we asked whether novel ER+ subgroups could be found by joint clustering of DNA methylation and gene expression data. Matched DNAm-mRNA data for 463 ER+ breast cancers from the TCGA and for all FEM genes which were either significantly differentially methylated or differentially expressed, or both, were used as input to a joint latent variable model for integrative clustering using the *iCluster* R package [17]. According to the proportion of deviance (POD) score [17], we obtained an optimal clustering solution at *k* = 2, i.e. there was no statistical evidence for more than two clusters. We observed that the two main clusters were strongly correlated with luminal subtype status (Fisher test *P* < 10^−10^, Fig. 3), indicating that a proportion of the transcriptomic differences between these two main ER+ subtypes is driven by underlying differences in DNA methylation. Importantly, we observed that luminal-B tumours showed higher levels of DNA methylation deviation from the normal samples, compared to luminal-A tumours, a trend which was also seen at the level of gene expression (Fig. 3). The far majority of the FEM genes exhibited low methylation levels in normal tissue, intermediate levels in luminal-A tumours and the highest levels in luminal-B’s. Correspondingly, gene expression levels were lower in luminal-A and lowest in luminal-B tumours (Fig. 3).

The homogeneity of the integrative DNAm mRNA expression landscape led us to investigate if this was the result of focusing on only significant genes within FEM modules. To address this, we reapplied the iCluster algorithm but now on the full set of genes exhibiting significant differential methylation and expression between normal and ER+ breast cancer. Specifically, we identified a total of 4311 genes exhibiting an anti-correlative relation between differential methylation and differential expression. Application of iCluster to these 4311 genes resulted once again in an optimal two-cluster solution, which also correlated strongly with luminal subtype status (Additional file 1: Figure S3). Thus, the lack of epigenetic substructure within the luminal subtypes is not an artefact of “casting a lower net” by the use of a PPI network in the FEM algorithm but instead indicates that putative DNAm-driven gene expression changes are surprisingly homogeneous, constituting a remarkably good discriminator of luminal-A/B subtype status. Indeed, based on the original clustering result over the FEM genes (Fig. 3), the luminal-A/B classification accuracy was as high as 0.75, with an adjusted Rand index (ARI) of 0.26. This contrasts quite significantly with integrative clustering analyses over copy number-driven gene expression changes, which in the METABRIC set resulted in as many as 10 intrinsic clusters [4, 5]. In fact, the highly heterogeneous nature of copy number-driven gene expression changes resulted in an adjusted Rand index of only 0.07 (as calculated over ER+ luminal subtypes only). However, we note that specific intrinsic cluster (IC) combinations (e.g. IC1 vs IC3) could achieve higher ARI values than those obtained from the integration of DNAm and mRNA expression (Additional file 1: Table S3).

### Luminal-A and luminal-B breast cancers are epigenetically similar but with the luminal-B subtype exhibiting larger deviations in DNA methylation

**Fig. 4:**
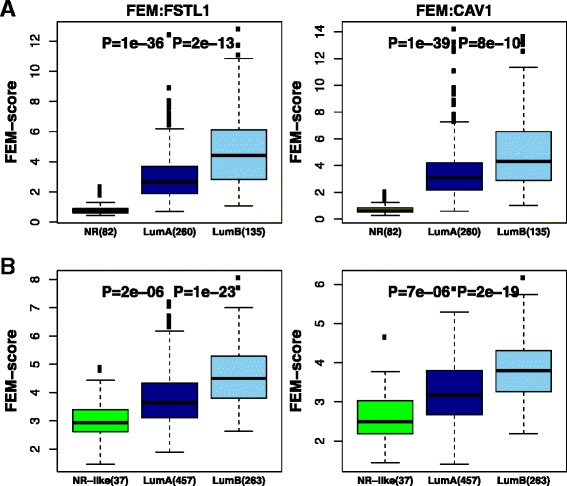
FEM modules discriminate normal, luminal-A and luminal-B breast cancers. **a** Boxplots of FEM deviation scores for the *FSTL1* and *CAV1* FEMs, stratified according to normal, luminal-A and luminal-B breast cancers. Wilcoxon rank-sum test *P* values between normal and luminal-A, as well as between the two luminal subtypes are shown. **b** Validation of (**a**) in the METABRIC ER+ set

To quantify the previous patterns of epigenetic deregulation more rigorously, we devised for each FEM module a FEM “deviation” score, which measures the deviation of DNAm and mRNA expression of a cancer sample relative to the normal samples (Methods). Confirming the previous result, all FEM modules exhibited higher deviation scores in luminal-B compared to luminal-A breast cancer (Fig. 4a and Additional file 1: Figure S4). We were able to validate this result, for instance using the METABRIC expression data set: despite not having DNA methylation data for these samples, the FEM deviation scores derived from only using gene expression data were always higher in luminal-B breast cancers, validating our previous finding (Fig. 4b and Additional file 1: Figure S5).

Importantly, we observed that the FEM deviation scores were also significantly higher in luminal-A breast cancer compared to normal breast tissue (Fig. 4 and Additional file 1: Figure S4), a clear indication that luminal-A and luminal-B breast cancers are epigenetically similar entities, with the only difference being the larger deviations seen in the luminal-B subtype. To confirm this further, we posited that the same FEMs would be retrieved had we run FEM using only luminal-A or luminal-B breast cancers. We therefore repeated the FEM analysis but now comparing only ER+ luminal-B cancers to normal tissue and then again comparing ER+ luminal-A’s to normals. Confirming the epigenetic similarity of both luminal subtypes of breast cancer, highly overlapping, if not identical FEMs, were retrieved in both separate analyses (Additional file 1: Table S4).

### Prognostic FEM modules in ER+ breast cancer and their correlation to proliferation

Next, we decided to study the prognostic significance of the identified FEM modules. For the two predicted clusters (Fig. 3), we constructed representative centroids, separately for DNAm and mRNA expression. Independent samples can then be classified into one of the two integrative subtypes by a nearest-centroid criterion rule (Methods). This procedure was applied to three independent data sets, including the METABRIC mRNA expression set [4], an independent Illumina 450K DNA methylation set of breast cancers (“Germany” set) and a third set (“Fleischer”) with matched 450K DNA methylation and gene expression data [9]. As a benchmark, we compared the prognostic separability of the predicted clusters with the luminal-A/B subtype classification (Table 3, Additional file 1: Figure S6). As assessed in a meta-analysis using the combined Fisher test over three independent studies with either DNAm or mRNA data available, we observed that both the DNAm- and mRNA-based integrative cluster centroids were predictive of prognosis (Table 3, Additional file 1: Figure S6). Although the integrative cluster centroids did not outperform the existing luminal-A/B classification as a prognostic model, they did exhibit stronger consistency across studies as the luminal-A/B classification failed to predict prognosis in the smaller Fleischer set (Table 3, Additional file 1: Figure S6). Importantly, in all data sets, samples classified into the cluster exhibiting the higher deviation scores were associated with a poorer clinical outcome (binomial test *P* = 0.03).

**Table 3:** Univariate Cox regression survival analysis of the iCluster classification DNAm (iCC-DNAm) and mRNA (iCC-mRNA) centroids in ER+ breast cancer, as well as that of the luminal subtype classification (Lum-A/B)

	iCC-DNAm		iCC-mRNA		Lum-A/B	
	HR (95 % CI)	*P*	HR (95 % CI)	*P*	HR (95 % CI)	*P*
TCGA	1.83 ( 0.86–3.90)	0.112	1.83 (0.86–3.90)	0.112	2.68 (1.20–5.99)	0.012
METABRIC	NA	NA	1.45 (1.20–1.75)	0.0001	1.82 (1.49–2.21)	2e-9
Germany	2.31 (1.07–4.99)	0.028	NA	NA	NA	NA
Fleischer	2.38 (0.81–6.97)	0.103	2.03 (0.64–6.37)	0.217	0.97 (0.23–3.58)	0.961
Combined Fisher test *P*		0.013		0.0002		7e-9

*NA* not available due to missing information

Hazard Ratios (HR), 95 % confidence intervals (CI) and likelihood ratio test *P* values in each data set are given. The *P* values under a combined Fisher test is also given

**Fig. 5:**
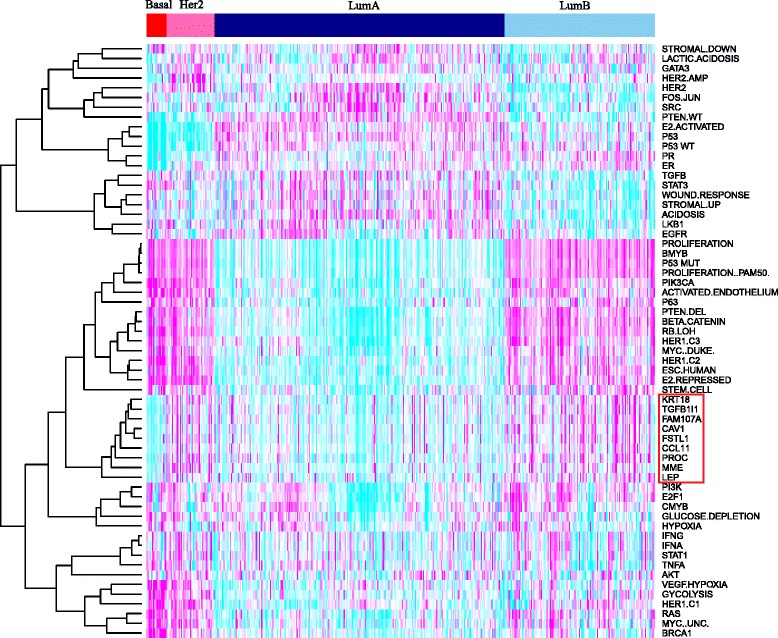
Clustering heatmap of the nine FEM deviation and 52 pathway activity scores. Hierarchical clustering analysis of 52 pathway activity scores from Gatza et al. (as derived in the TCGA ER+ data set) and the nine FEM scores. Colour codes: *cyan* = low relative activity and *magenta* = high relative activity. ER+ breast cancers have been ordered according to their PAM50 intrinsic subtype

To further assess the significance of the FEM modules, we clustered their FEM deviation scores in a clustering analysis which also included activity estimates of various signalling pathways and proliferation indices, as recently estimated by Gatza et al. [18]. All nine FEMs clustered together, revealing that their overall patterns of epigenetic deregulation are highly similar (Fig. 5). However, this clustering is also expected given that the signalling pathway activity estimates from Gatza et al. do not use epigenetic information. Of note however, the FEM modules clustered most closely with a cluster of biological pathways which included proliferation, *B-MYB* activation, *p53* mutation, *PIK3CA* activation and stemness, supporting their association with a highly proliferative and poor-outcome ER+ breast cancer (Fig. 5). Confirming this further, an overall FEM score computed over all significant FEM module genes correlated reasonably well with a single-gene proliferation index (*PCNA* expression) (Pearson correlation ∼0.38, *P* < 10^−10^).

### DNA methylation-associated expression changes are coordinated and not mutually exclusive

One of the most interesting insights to have emerged from TCGA studies is that functional cancer driver events targeting a common signalling pathway often occur in a mutually exclusive fashion within the same tumour [10]. We asked if the same might be true for DNA methylation changes. Specifically, it is reasonable to ask whether key genes within FEM modules exhibit mutual exclusive patterns or if instead they exhibit coordinated changes (i.e. changes happening within the same tumour). We note that because FEMs are inferred from statistics of differential DNAm and mRNA expression (which are derived from comparing phenotypes, i.e. from comparing all samples within respective phenotypes), there is no requirement for the changes within FEMs to be coordinated.

**Fig. 6:**
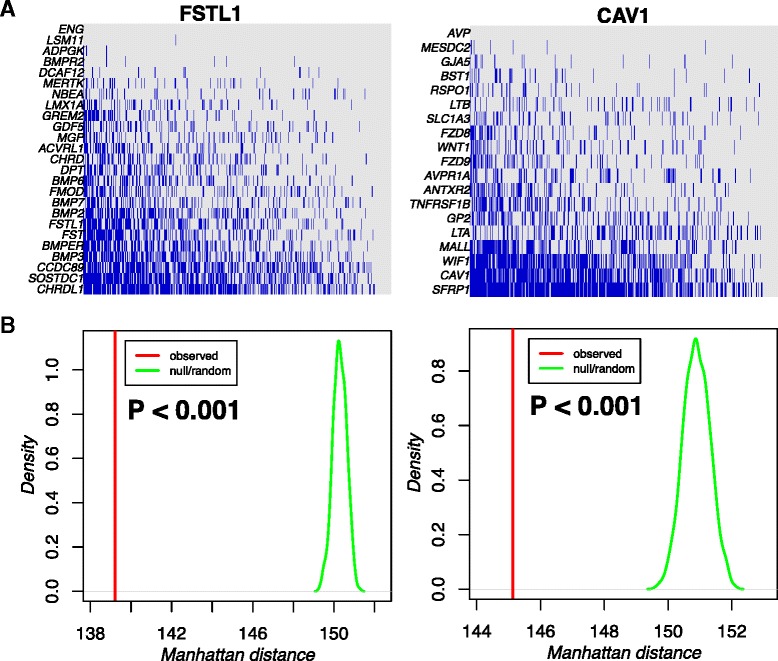
FEM module genes exhibit coordinated DNAm changes. **a** Binary matrix representation of two FEM modules, with *rows* labelling genes and *columns* labelling ER+ breast cancers. In the heatmap, *blue* indicates that the gene’s DNAm level in the given cancer sample deviates significantly from the normal tissue values. **b** Comparison of the observed average Manhattan distance between FEM module genes (*red vertical line*), as estimated from the binary matrix representations in (**a**), to the distribution of average Manhattan distances obtained by permuting the samples within each row (1000 permutations, *green density curves*). Empirical *P* value for the observed distance is given

To investigate whether patterns are coordinated or mutually exclusive, we constructed for each FEM module a binary representation matrix over the significantly differentially expressed and differentially methylated genes in the FEM and across all ER+ cancers (Methods). Each binary entry in this matrix indicates whether a given gene exhibits a significantly different DNAm and mRNA level (assuming anti-correlation between the two) from the corresponding normal tissue. We observed that different genes within the same FEM module exhibited a tendency for their DNAm and mRNA levels to deviate from the normal tissue in the same tumour samples (Fig. 6a, Additional file 1: Figure S7). This was confirmed by comparing the average Manhattan distance between pairs of genes to that obtained by permuting sample labels (Fig. 6b, Additional file 1: Figure S7).

Finally, to show that there is indeed a difference in the level of mutually exclusive patterns exhibited by DNA methylation and copy number, we compared the average path length distance of top-ranked differentially methylated genes to top-ranked amplified/deleted genes, using the TCGA data for which both DNA methylation and Affy SNP data is available. Focusing on the same number of top-ranked genes by differential methylation or copy number change, we observed that the average shortest path length distance between top-ranked differentially methylated genes was indeed smaller than for the correspondingly top-ranked amplified/deleted genes (Additional file 1: Figure S8).

## Discussion

Here, we have performed a detailed integrative analysis of DNA methylation and gene expression data in the context of ER+ breast cancer. We first performed this integrative analysis within a system-level framework, using the FEM algorithm to integrate both data types in the context of a comprehensive protein interactome, in an attempt to identify specific gene modules or signalling pathways which are functionally deregulated in ER+ breast cancer as a result of underlying changes in DNA methylation. This revealed a number of hotspots, four of which could be validated as hotspots in an independent (unmatched) DNA methylation and gene expression set. Remarkably, integrative clustering of DNA methylation and matched gene expression over the significant genes within these FEMs only revealed two main clusters. The same result was obtained after clustering over all genes exhibiting anti-correlative patterns between differential DNAm and mRNA expression, indicating that the relative homogeneity of the epigenetic landscape of ER+ breast cancer was not due to us using the FEM algorithm. Importantly, the two main clusters correlated very strongly with luminal-A/B subtype status. Indeed, our work clearly shows that whereas the luminal-B and luminal-A subtypes do not differ in terms of the specific FEMs (since for both subtypes, the FEM deviation scores are higher compared to normal tissue), they do however differ significantly in terms of the absolute levels of DNA methylation and gene expression deviation from the normal reference. Specifically, luminal-B cancers invariably showed higher levels of promoter DNA methylation and correspondingly lower levels of gene expression, than luminal-A cancers. Our result thus extends a previous observation by a recent study reporting higher levels of promoter DNAm in luminal-B breast cancer [19]: our study extends this to showing that mRNA expression deviations, driven putatively by underlying DNA methylation changes, are more marked in luminal-B breast cancer compared to luminal-A’s. In other words, the data presented here reveals that both luminal subtypes are characterized by the same epigenetic driver patterns, only differing in the *level* of deregulation.

The relative homogeneity of the integrative epigenomic-transcriptomic landscape of ER+ breast cancer contrasts with the corresponding integrative landscape at the copy number level. Indeed, the recent METABRIC study, which performed an integrative clustering of copy number and transcriptomic changes for genes exhibiting significant correlations, revealed considerable heterogeneity with at least 10 integrative subtypes across all breast cancers. An implication of this, as we have seen, is that unsupervised clustering of DNA methylation-induced gene expression changes appears to discriminate luminal-A from luminal-B tumours with a much higher accuracy than what appears possible based on copy number data. This is interesting because it suggests that DNA methylation changes explains much more of the homogeneity in the transcriptomic differences observed between luminal-A and luminal-B breast cancers, whereas copy number changes may explain more of the heterogeneous differences. Thus, from a purely epigenetic perspective, our data suggests that ER+ breast cancer consists of only two main epigenetic subtypes, which differ mainly in the level of epigenetic deregulation, and not in the specific deregulated genes or signalling pathways.

Illustrating this surprising homogeneity is a FEM module, centred around *CAV1* and which was strongly enriched for many members of the WNT signalling pathway. Many members of this module, including *WIF1*, *WNT1*, *SFRP1*, *FZD9*, and *FZD8* were hypermethylated and underexpressed in both luminal-A and luminal-B breast cancers but exhibiting larger deviations in DNAm and mRNA expression in the luminal-B subtype. One potential interpretation of this is that luminal-A and luminal-B breast cancers are epigenetically similar entities but with the higher proliferation rate of luminal-B cancers causing increased deviations from the normal epigenetic landscape. The moderate significant correlation of the FEM module score with the proliferation marker *PCNA* partially supports this. On the other hand, the WNT signalling pathway has been implicated as a key pathway underlying breast cancer development and progression [20–27]. Indeed, DNA methylation-induced silencing of key WNT signalling antagonists is thought to be the prime mechanism causing nuclearization of beta-catenin and an increased cell proliferation rate [21–23]. In the context of a cancer stem cell model, such overactivity of WNT signalling may increase self-renewal at the expense of differentiation, an imbalance which may promote tumourigenesis [22, 28]. Our data fully supports these models, as we also see epigenetic silencing of many WNT signalling antagonists, including notably *WIF1* [20] and *SFRP1* [28], the latter also being an unfavourable prognostic marker [27]. Thus, epigenetic silencing of WNT signalling antagonists could lead to higher activity of this pathway, increasing self-renewal at the expense of differentiation, as envisaged by Baylin and Ohm [28]. Interestingly, the WNT signalling pathway was also found to represent an epigenetically deregulated hotspot in the context of ageing, independently of tissue type [13]. Hypermethylation of WNT antagonists has also been shown to correlate with a patient’s age [26]. Thus, epigenetically induced hyperactivity of this pathway could reflect an early event in the pathogenesis of ER+ breast cancer.

Besides WNT signalling, another important FEM mapped to the TGF-β/BMP signalling pathways. Among the 18 genes which mapped to the WNT signalling pathway, 7 exhibited simultaneous hypermethylation and underexpression. Among these, FST and FSTL1 are known to bind activin members of the TGF-beta superfamily and function to control cellular proliferation [29]. Hypermethylation of FST/FSTL1 could directly impair this restraint, leading to cancer progression. Supporting this, *FSTL1* appears to play a tumour suppressor role in two other hormone-related women cancers (ovarian and endometrial cancer) [29].

Many bone morphogenetic proteins, including BMP2, BMP6, and BMP7, were also simultaneously hypermethylated and underexpressed in the same FEM *FSTL1* module, whereas TGF-β family members TGFB1 and TGFB3 were significantly overexpressed. Interestingly, a recent study showed that BMP7 significantly inhibited the TGF-β1-activated epithelial-mesenchymal transition (EMT)-related genes in breast cancer cells, resulting in a significant reduction in TGF-β1-triggered cell growth and cell metastasis, suggesting that the BMP7 signalling axis could be a promising pathway for therapeutic intervention in breast cancer [30]. BMP6 has also been identified as a potential tumour suppressor associated with differentiation and metastasis [31–36], which reverses EMT in breast cancer by restoring E-cadherin expression [32, 36]. Epigenetic silencing of BMP2 in breast cancer has also been found to promote breast cancer progression and drug resistance, as well defining a novel prognostic marker and offering novel therapeutic opportunities [37].

Among the other inferred FEM modules, it is worth highlighting the following. The *CCL11* FEM module was significantly enriched for chemokines and chemokine receptors, which were mostly hypomethylated and overexpressed. Chemokines have been reported to influence the metastatic potential of breast cancer [38]. Interestingly, the *LEP* FEM module contained several G protein-coupled receptors (GPCRs), which could play a role in mediating the effects of chemokines. The *MME* FEM module was significantly enriched for genes in endothelin pathways. Previous research has found that endothelin signalling plays a crucial role in cell differentiation, proliferation and migration processes [39]. Consistent with previous findings, we found *EDN2* to be hypomethylated and overexpressed, whereas *EDN3* was hypermethylated and underexpressed. Finally, the *PROC* FEM module was significantly enriched for genes involved in the fibrin clot clotting cascade. Coagulation and fibrinolysis are known to serve as haemostatic elements that can facilitate the metastatic potential of breast cancer [40]. In summary, the fact that the FEM algorithm has retrieved many diverse signalling pathways with important roles in breast cancer progressions attests to the power and usefulness of this algorithm.

Another interesting observation of our study is the absence of a mutually exclusive pattern of DNA methylation changes within FEM modules. Whereas copy number changes within signalling pathways have been shown to occur in a mutually exclusive fashion [10], the same does not appear to hold for DNA methylation. This suggests that aberrant DNA methylation in cancer is characterized by a modularity in the sense that many components of the same gene module or pathway may be affected in the same sample. This may not be that surprising if one realizes that DNA methylation changes in development exhibit a form of modularity. As concrete examples, we have previously observed that DNA methylation patterns exhibit a level of correlative modularity (in the context of PPI networks) in normal tissue [13], as well as observed differentially methylated gene modules underlying endothelial cell differentiation [11]. Moreover, a key feature of development is alterations of DNA methylation at binding sites of tissue-specific transcription factors [41], which means that common gene targets (which are also likely to be part of the same pathway) will exhibit similar DNAm patterns in the same sample. Thus, there is ample data supporting the view that in cancer, we would also observe modularity of DNAm changes at the single-sample level.

The recent observation that DNAm changes may mediate functional changes primarily by reorganizing the patterns of transcription factor binding [41] also raises an important caveat to our analysis. We focused specifically on correlations between proximal gene promoter DNAm and expression. Thus, while our analysis supports the view that this specific integrative epigenetic-transcriptomic landscape is fairly homogeneous in ER+ breast cancer, this does not by any means exclude the possibility that ER+ breast cancer is highly heterogeneous in relation to the correlative patterns of DNA methylation at distal regulatory elements and downstream gene expression. Further investigation of the integrative epigenetic-transcriptomic landscape including distal enhancer-promoter interactions is warranted.

## Conclusions

In summary, our work demonstrates that a key component of the integrative epigenomic-transcriptomic landscape of ER+ breast cancer is surprisingly homogeneous. Specifically, we have shown that from the perspective of proximal promoter DNA methylation changes that drive gene expression alterations, luminal-B and luminal-A are characterized by the same epigenetically deregulated hotspots but with luminal-B tumours exhibiting more aggravated levels of DNA methylation and gene expression change from the normal state. Our data point towards WNT and BMP signalling as key epigenetically deregulated pathways underlying both luminal subtypes, specially luminal-B breast cancers.

## Methods

### Gene expression data sets

#### TCGA

Illumina HiSeq 2000 RNA Sequencing Version2 Level 3 data for breast invasive carcinoma samples was acquired from the TCGA data portal. Samples were filtered to include only 724 ER-positive breast cancers and 111 adjacent normal breast tissue samples. RNA-Seq data included gene-normalized values for 20,531 genes. Normalized values equal to 0 were substituted by the minimum non-zero positive value. Values were then log2-transformed in order to regularize the dynamic range. Inter-array normalization using the quantile method was then performed using the *limma* package [42].

#### Yu

Expression data for 13 normal breast tissue samples and 110 ER-positive breast cancer samples encompassing 13,262 genes was obtained from Yu et al. [43].

#### METABRIC

Normalized Illumina HT-29 v3 expression data of the METABRIC project (*n* = 1992 samples) were acquired from the European Genome-phenome Archive at the European Bioinformatics Institute. We used only the 774 ER-positive breast cancer samples from the METABRIC discovery data set, given that the validation set exhibited significantly lower cellularity. The expression data matrix included 24,924 genes.

#### Fleischer

Expression data of the study of Fleischer et al. [9] was downloaded from GEO (accession number GSE19783). The data matrix consisted of 104 breast cancer samples and 19,596 genes.

### DNA methylation data sets

#### TCGA

Illumina Infinium Human Methylation 450K level 3 DNA methylation data for human breast cancer samples was acquired from the TCGA data portal. Samples were filtered to include only 471 ER-positive breast cancers and 96 adjacent normal breast tissue samples. We removed low-quality probes which lacked beta values for more than half of the samples. The rest of the missing values were imputed with the R package *impute* using *k* = 5 [44], resulting in a data matrix of 395,775 CpG probes.

#### Germany

Illumina 450K DNA methylation data for 49 normal breast tissue samples and 254 ER-positive breast cancer samples was done from samples collected within the Bavarian Breast Cancer Cases and Controls Study 2. The Ethics Committee of the Medical Faculty, Friedrich-Alexander University, approved the study (Re. No. 4514), and all patients gave written informed consent. Data are available on GEO under accession number GSE69914. Data underwent a quality control procedure as implemented by us previously [45]. This resulted in a fully normalized data matrix for 485,512 CpG probes.

#### Fleischer

Illumina 450K methylation data from [9] were downloaded from GEO (same accession as gene expression data), for a total of 285 fresh frozen tissue samples. Data underwent quality control encompassing 468,424 CpG probes. The samples included 46 normal breast tissue samples from healthy women, 22 pure DCIS, 31 mixed DCIS-IBC and 186 IBC of stage I and II. Of these, 104 IBC samples had matched expression data.

### Brief review of the FEM algorithm

The Functional Epigenetic Module (FEM) algorithm is a functional supervised algorithm which is aimed at identifying functionally related genes which exhibit both differential DNA methylation and differential expression in relation to some phenotype of interest [11, 12]. The algorithm consists of three main parts: (1) assignment of differential DNAm and mRNA expression statistics for each gene; (2) integration of these statistics with a protein-protein interaction (PPI) network, whereby the edges of the network are assigned weights based on a function of the statistics of association for the two genes making up the edge; and (3) inference of hotspots of differential methylation and differential expression, by identifying subnetworks for which the average weight density (called modularity) is maximized locally relative to that predicted by a null distribution.

Because the statistics of differential DNA methylation are constructed at the gene level, there are many possibilities on how to do this in the case of the Illumina 450K beadarrays as there are many probes mapping to a given gene. As shown in Jiao et al. [11], probes mapping to the TSS200 region of a gene are in general the most predictive of gene expression, and moreover for such probes, high levels of DNAm are generally associated with underexpression. We therefore assign to each gene the average DNAm value of probes mapping to within 200 bp of the TSS. However, not all genes have probes mapping to their TSS200 region. For these genes, we use instead the average of probes mapping to the first exon of the gene, which as shown in Jiao et al. is the second most predictive region of gene expression. For genes with no probes mapping to TSS200 or first exon regions, we use the average of probes mapping to within 1500 bp of the TSS (the third most predictive region). Probes mapping to the gene body are not used. For each gene *g*, we thus obtain two statistics of association, one at the DNAm level (*t*_*g*_^(*D*)^) and another at the mRNA level (*t*_*g*_^(*R*)^). The statistics are inferred using an empirical Bayesian method [42].

We then assign to any given edge in the network, specified by genes *g* and *h* an average integrated statistic according to the following rule:

1 $$ \begin{array}{c}{t}_g^{(I)}=\left\{H\left({t}_g^{(D)}\right)H\left(-{t}_g^{(R)}\right)+H\left(-{t}_g^{(D)}\right)H\left({t}_g^{(R)}\right)\right\}\left|{t}_g^{(D)}-{t}_g^{(R)}\right|\\ {}{w}_{gh}=\frac{1}{2}\left({t}_g^{(I)}+{t}_h^{(I)}\right)\end{array} $$

where *H*(*x*) defines the Heaviside function.

Inference of hotspots/modules on the maximally connected subnetwork of the full PPI then proceeds using a spin-glass algorithm, as described in detail in [13]. Very briefly, we implement a local greedy version of the spin-glass algorithm, which aims to identify modules around each of the top-ranked differentially methylated and expressed genes. Significant modules are obtained using two complementary significance tests, one which uses the topological structure of the network into account and another which does not (this one randomizes the statistics over the network) [13].

### Identification of FEMs in ER+ breast cancer

We performed a system-level integrative analysis of DNA methylation and gene expression data using our previously published FEM algorithm [11, 12], in order to identify gene modules and signalling pathways which are functionally disrupted in ER+ breast cancer through aberrant DNA methylation. For discovery, we used the Illumina 450K DNAm and RNA-Seq TCGA data. The TCGA data set consisted of 567 samples for DNA methylation (96 normal and 471 ER positive) and 835 samples for mRNA expression (111 normal and 724 ER positive) encompassing 395,775 CpGs and 20,531 genes. To perform the system-level integration, we used the PPI network from our previous works (see e.g. [11, 12]). This resulted in X genes common across the three data types and inference of interactome hotspots of differential methylation and expression proceeded on a connected subnetwork of the PPI of Y genes. The statistics used in the FEM algorithm to weight the edges in the network were derived by comparing the DNAm and mRNA profiles of normal tissue to the ER+ breast cancer samples, using the empirical Bayes method as implemented in the *limma* package. Statistics of one data type (mRNA) were then scaled to ensure equal variance of statistics of both data types, as described previously [11, 12].

### Validation of FEMs

As validation set, we used the Illumina 450K methylation data (Germany) and Affymetrix mRNA expression data from Yu et al. [43]. These include 303 samples for methylation (49 normal and 254 ER positive) and 123 samples for expression (13 normal and 110 ER positive) encompassing 485,512 CpGs and 13,262 genes, respectively. In order to validate inferred FEMs, we followed the same procedure as described in [13]. Briefly, to validate the hotspot nature of the FEMs, the differential methylation and differential expression statistics derived in the new data sets were used to weight the network. Modularity values for the previously inferred FEMs were calculated and significance estimates obtained by random permutation (a total of 1000 permutations) of node statistics over the network. Significance *P* values in the validation set were obtained for each FEM by counting the number of permutations where the modularity score (average edge weights of the FEM module) is larger than the observed one.

The second validation compares the differential methylation and expression statistics between discovery and validation sets for all genes within a FEM module. A scatterplot of these statistics should yield a significant positive correlation. This validation tests for consistency in the directional patterns of change but does not assess the hotspot nature of the FEM modules in the validation set.

### iCluster

Matched DNAm and mRNA data for 463 ER+ breast cancers from the TCGA and for all FEM genes which were either significantly differentially methylated or differentially expressed, or both (241 genes), were used as input to a joint latent variable framework for integrative clustering using the *iCluster* R package [17]. The optimal clustering solution was obtained by examining the proportion of deviance (POD) score for different pre-specified numbers of clusters *k* (*k* = 2, 3, 4, 5, 6) and for different values of the sparsity parameter *λ* (*λ* = 0, 0.01, 0.05, 0.1, 0.15). The optimal clustering solution was obtained at POD = 0.0086 corresponding to *k* = 2 and *λ* = 0.1.

### Constructing the FEM score

In order to be able to quantify the deviation in DNAm and mRNA expression of FEM module genes from the values in the normal reference tissue, we devised for each module a sample-specific FEM score. Construction of this score requires samples with both DNAm and mRNA expression data, so this score computation was restricted to the matched 82 normal samples and 463 ER-positive breast cancers. Deviations were calculated using the mean and standard deviation of each gene across normal samples. Given a FEM module, we only use those genes which gave rise to the significance of the module in the first place, i.e. we include only genes that are significant at both differential DNAm and differential mRNA levels, and which also exhibit an anti-correlation (i.e. hypermethylated and underexpressed or hypomethylated and overexpressed in ER+ breast cancer). Thus, let *g* denote such a gene and *s* denote a tumour sample. We then compute *Z*-statistics as

$$ {Z}_{gs}^{(D)}=\frac{X_{gs}^{(D)}-{\mu}_g^{(D)}}{\sigma_g^{(D)}} $$

$$ {Z}_{gs}^{(R)}=\frac{X_{gs}^{(R)}-{\mu}_g^{(R)}}{\sigma_g^{(R)}} $$

where *D* and *R* stand for DNA methylation and mRNA expression, respectively, and where *μ* and *σ* denote the mean and standard deviation, respectively, as estimated over the normal samples. A complication arises from the fact that the two *Z*-statistics are not directly comparable due to intrinsic factors related to each data type. Thus, when constructing an integrated score, in order to avoid biasing the score to one data type, we need to introduce a scaling factor *α*, which will be data-type specific. To estimate this scaling factor, we compute the standard deviation of DNAm and mRNA *Z*-statistics over all genes (i.e. not just FEM genes) and samples, quantities we denote by *σ*_*Z*_(*D*) and *σ*_*Z*_(*R*). We then define the scaling factor as the ratio of the standard deviations obtained from the DNA methylation and mRNA expression *Z*-statistics,

2 $$ \alpha =\frac{\sigma_Z(D)}{\sigma_Z(R)} $$

Finally, the FEM score of the FEM module in sample *s* is calculated as

3 $$ {FEM}_S=\frac{1}{m}{\displaystyle {\sum}_g^m}\left|{Z}_{gs}^{(D)}-\alpha {Z}_{gs}^{(R)}\right| $$

where *m* is the number of genes in the FEM module passing the requirements mentioned above.

### Construction of iCluster centroids and classification

The iCluster algorithm over all significantly anti-correlated FEM genes (99 genes in total) in ER+ breast cancer resulted in only two clusters. Centroids at the DNAm and mRNA level were constructed by averaging the corresponding values of the samples in each cluster. For each gene, its DNAm and mRNA profile was centred before performing the averaging per cluster. With the centroids thus defined, i.e. two centroids for each of the DNAm and mRNA data, independent ER+ samples can then be classified using a nearest-centroid classification rule. Specifically, for an independent ER+ mRNA or DNAm data set, each of the 99 genes was first centred to mean zero. Samples were then classified using a nearest-centroid criterion, with a Spearman rank correlation coefficient defining the distance metric. For data sets with matched DNAm and mRNA data (i.e. Fleischer set), we thus obtained two independent classifications, one from the DNAm and another from the mRNA data.

### Coordinated vs mutually exclusive patterns

#### FEM modules

To determine whether DNAm-associated mRNA expression changes of FEM genes within FEM modules occur in a coordinated or mutually exclusive manner within individual tumour samples, we first constructed a binary representation matrix for each FEM module using only genes that are differentially methylated and differentially expressed and which exhibit an anti-correlative pattern. The binary matrix was obtained by calculating *Z*-statistics for each gene in each tumour sample, by comparing the DNAm and mRNA level in the sample to the normal tissue samples. Specifically, we estimated the mean and variance for each gene across the normal samples and then computed the *Z*-statistic deviation score. Assuming a Gaussian distribution for the normal samples, we thus obtained two *P* values (one for DNAm and another for mRNA expression) for each gene in each tumour sample. We then assigned a value of 1 to the corresponding matrix entry if both *P* values were less than 0.1 and if there is an anti-correlative pattern (i.e. hypermethylated and underexpressed or hypomethylated and overexpressed); otherwise, we assigned a value of 0. The relaxed threshold was chosen because (i) the FEM algorithm infers modules without imposing specific thresholds on the statistics of differential expression and methylation and (ii) this allowed us to assess the patterns in more genes within FEMs. For each FEM, we next estimated the average Manhattan distance (MHD) between the rows (significant genes) of this binary matrix. This distance was then compared to the distance obtained by independently permutating the samples within each row, using a total of 1000 permutations to derive a null distribution. An overall *P* value was then obtained by counting the number of permutations with an average MHD more extreme than the observed one.

#### Comparison to copy number

To objectively assess the level of coordination or mutual exclusivity of DNAm changes, we decided to compare these changes to those at the copy number level. Thus, we compared the distribution of the shortest paths of top-ranked differentially methylated genes to that of the top-ranked amplified/deleted genes, estimated in the context of the same PPI network used in the FEM algorithm. However, genes were ranked according to differential DNA methylation between normal and ER+ breast cancer without using FEM. We selected the top 100 and top 200 ranked differentially methylated genes, as well as the top-ranked 100 and 200 genes according to copy number change using the matched TCGA breast cancer data. The shortest paths were then estimated between every pair of genes in the top-ranked list, and both the distribution and average compared between DNAm and copy number.

## Additional file

Additional file 1:
**Supplementary figures and tables**. Contains Figures S1–S8 and Tables S1–S4. (PDF 2191.36 kb)

## Abbreviations

DNAm: DNA methylation
ER: estrogen receptor
FEMs: Functional Epigenetic Modules
PPI: protein-protein interaction
TCGA: The Cancer Genome Atlas
