# Erratum to: The integrative epigenomic-transcriptomic landscape of ER positive breast cancer

**DOI:** 10.1186/s13148-016-0231-4

**Published:** 2016-06-01

**Authors:** Yang Gao, Allison Jones, Peter A. Fasching, Matthias Ruebner, Matthias W. Beckmann, Martin Widschwendter, Andrew E. Teschendorff

**Affiliations:** CAS Key Lab for Computational Biology, CAS-MPG Partner Institute for Computational Biology, Chinese Academy of Sciences, Shanghai Institute for Biological Sciences, 320 Yue Yang Road, Shanghai, 200031 China; Department of Women’s Cancer, University College London, 74 Huntley Street, London, WC1E 6BT UK; Department of Gynaecology and Obstetrics, Friedrich-Alexander University Erlangen-Nuremberg, University Clinic Erlangen, Erlangen, 91054 Germany; Statistical Genomics Group, Paul O’Gorman Building, UCL Cancer Institute, University College London, 72 Huntley Street, London, WC1E 6BT UK

## Erratum

Unfortunately, after publication of the original version of this article [1], we noticed an error in Figure S8 in Additional file 1. Specifically, the error relates to the legend of the figure, as what is being diplayed in this figure is not the shortest path between pairs of top ranked features, but the smallest number of path links one needs to remove in order for the pair of features to be disconnected. Unfortunately, this completely alters the corresponding statement in the last paragraph of the Results section, since the smaller average path link number for DNA methylation, as shown in Figure S8, means that in effect top ranked DNA methylation alterations occur more distantly apart on the network than top-ranked copy-number alterations. This does not however alter the conclusion about DNA methylation exhibit coordinated changes across samples, as shown in Fig. 6, since the analysis presented in Figure S8 does not consider alterations in individual samples, whereas the data shown in Fig. 6 does. Hence, the main conclusion that DNA methylation alterations in FEM modules exhibit coordination modularity is unaffected. However, top-ranked DNA methylation alterations map further apart on the network than corresponding copy-number alterations.
