# Supplementary material for: The integrative epigenomic-transcriptomic landscape of ER positive breast cancer
Source: Clin Epigenetics. 2015 Dec 9;7:126. doi: 10.1186/s13148-015-0159-0 (PMC4673726; doi:10.1186/s13148-015-0159-0)
Supplement: Additional file 1: — Supplementary figures and tables. Contains Figures S1–S8 and Tables S1–S4. (PDF 2191.36 kb) [file 13148_2015_159_MOESM1_ESM.pdf]

Additional File 1 for:

The integrative epigenomic-transcriptomic landscape of ER positive breast cancer

Yang Gao[1] , Allison Jones[2] , Peter A. Fasching[3], Matthias Ruebner[3], Matthias W. Beckmann[3], Martin Widschwendter[2] and Andrew E. Teschendorff[1,2,4*]

[1] CAS Key Lab for Computational Biology, CAS-MPG Partner Institute for Computational Biology, Chinese Academy of Sciences, Shanghai Institute for Biological Sciences,320 Yue Yang Road, 200031 Shanghai, China. [2] Department of Women's Cancer, University College London, 74 Huntley Street, London WC1E 6BT, UK. [3] University Clinic Erlangen, Department of Gynaecology and Obstetrics, Friedrich-Alexander University Erlangen-Nuremberg, Erlangen, Germany. [4] Statistical Genomics Group,Paul O'Gorman Building, UCL Cancer Institute, University College London, 72 Huntley Street, London WC1E 6BT, United Kingdom.

*Corresponding Author: a.teschendorff@ucl.ac.uk, andrew@picb.ac.cn

SUPPLEMENTARY FIGURES:

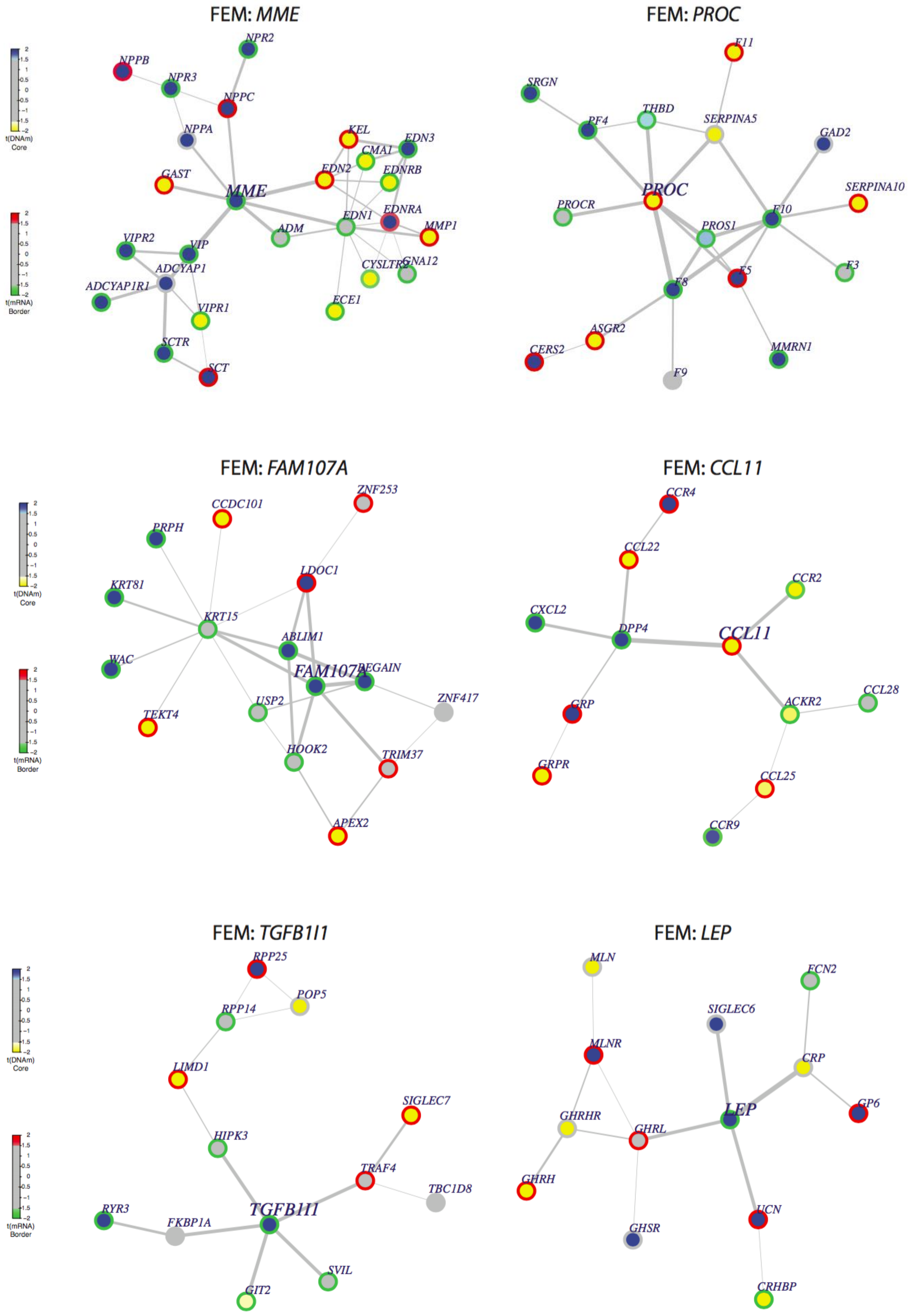

t(DNAm) Core
t(mRNA) Border
FEM: MME
NPR2
NPPB
NPR3
NPPC
NPPA
GAST
MME
KEL
EDN3
CMA1
EDN2
EDNRB
EDNRA
EDN1
MMP1
ADM
VIPR2
VIP
ADCYAP1
ADCYAP1R1
CYSLTR2
GNA12
ECE1
VIPR1
SCTR
SCT
FEM: PROC
F11
SRGN
THBD
PF4
SERPINA5
GAD2
PROC
SERPINA10
PROCR
F10
PROS1
F5
F3
F8
ASGR2
CERS2
MMRN1
F9
FEM: FAM107A
ZNF253
CCDC101
PRPH
LDOC1
KRT81
KRT15
ABLIM1
WAC
FAM107A
BEGAIN
USP2
ZNF417
TEKT4
HOOK2
TRIM37
APEX2
FEM: CCL11
CCR4
CCL22
CCR2
CXCL2
DPP4
CCL11
CCL28
GRP
ACKR2
GRPR
CCL25
CCR9
FEM: TGFB1I1
RPP25
POP5
RPP14
LIMD1
SIGLEC7
HIPK3
TRAF4
TBC1D8
RYR3
FKBP1A
TGFB1I1
SVIL
GIT2
FEM: LEP
MLN
FCN2
SIGLEC6
MLNR
CRP
GP6
LEP
GHRHR
GHRL
GHRH
UCN
GHSR
CRHBP

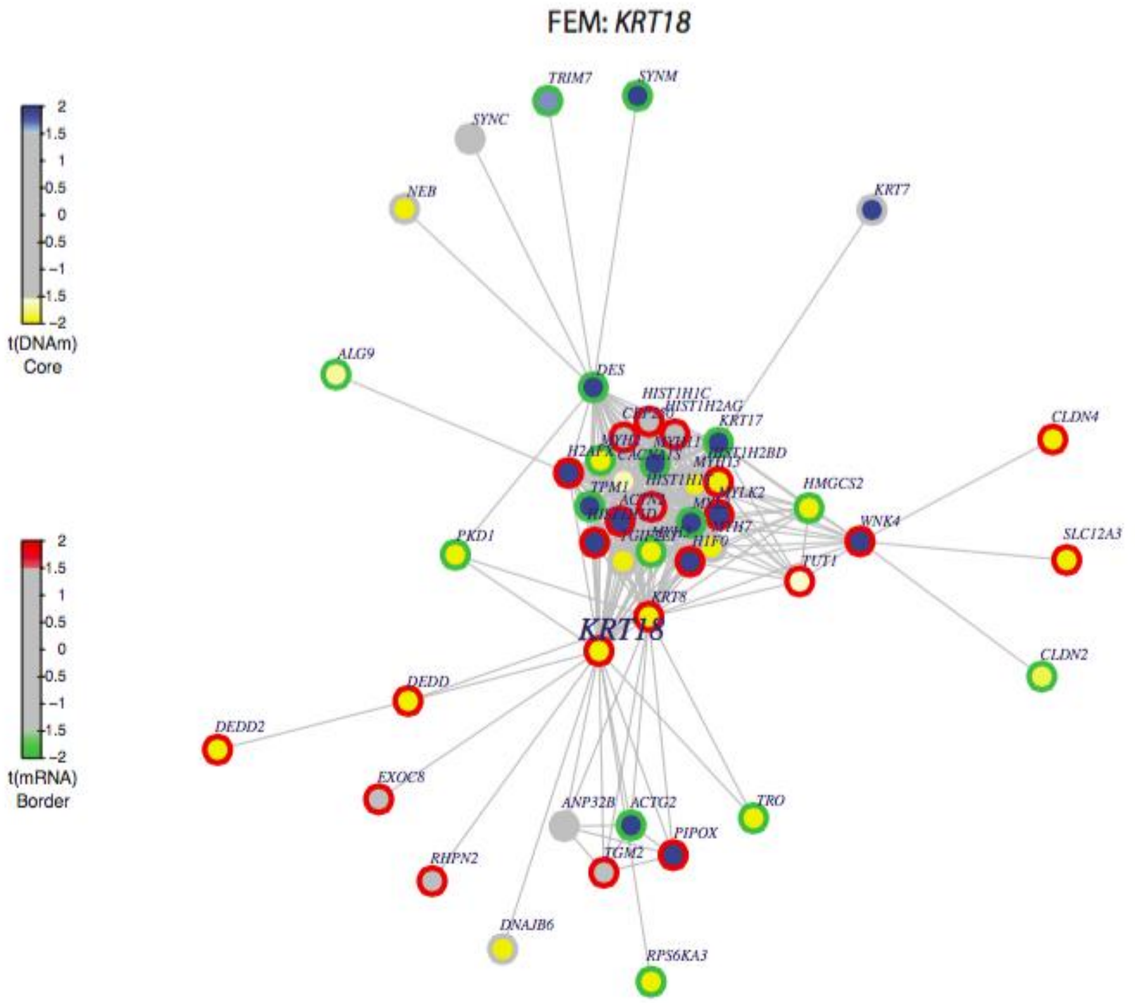

Fig.S1: Network representations of 7 of the 9 inferred FEM modules in ER+ breast cancer (the other 2 modules are shown in Fig.1B). Colors indicate directionality and significance of differential DNAm and mRNA expression, as shown, i.e. blue (yellow) nodes indicate promoter hypermethylation (hypomethylation) in cancer vs. normal, whereas red (green) borders indicates overexpression (underexpression) in cancer vs. normal.

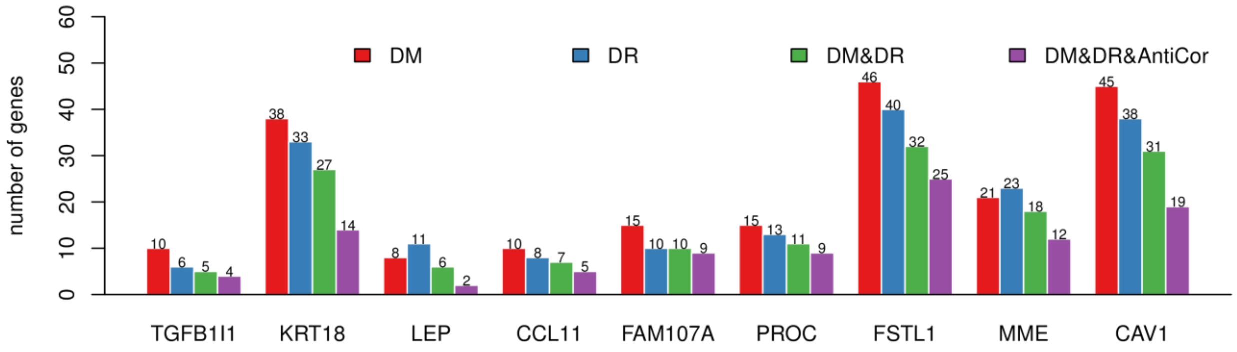

Fig.S2: For each FEM-module we give the numbers of differential methylated genes (DM), the number of differentially expressed genes (DR), the number of differentially methylated and differentially expressed ones (DM&DR) and the number of these which exhibited the anti-correlation between promoter differential methylation and differential expression (DM&DR&AntiCor). DM: significantly differentially methylated genes between normal and ER+ breast cancer samples found by computing moderated t statistics using empirical Bayes moderation of the standard errors and selecting those with p values < 0.05. DR: significantly differentially expressed genes between normal and ER+ breast cancer samples found by computing moderated t statistics using empirical Bayes moderation of the standard errors and selecting those with p values < 0.05.

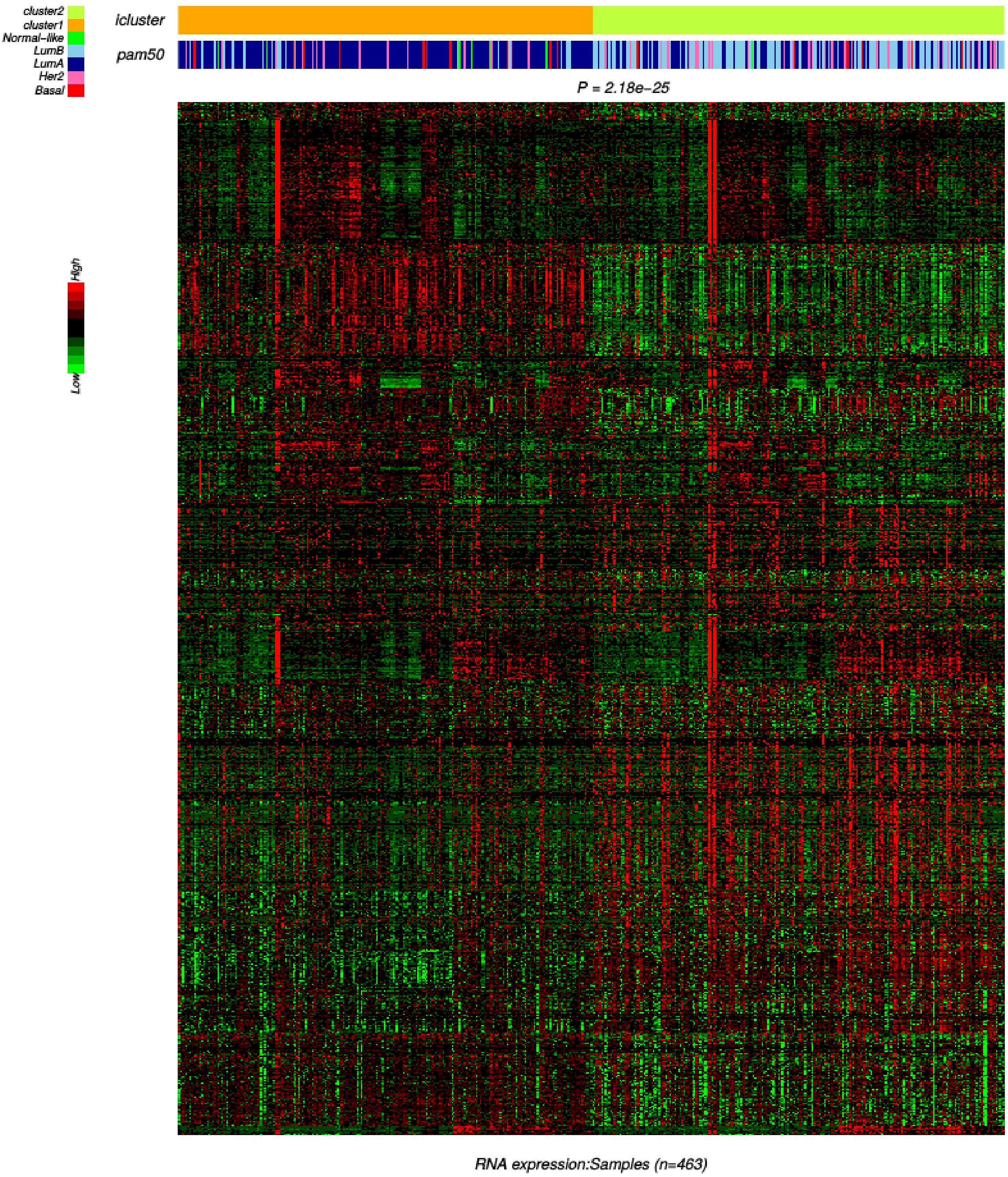

Fig.S3-partA: Integrative clustering of matched DNAm and mRNA data from 463 ER+ breast cancers using 4311 genes exhibiting an anti-correlative relation between differential methylation and differential expression (between normal and ER+ breast cancer). Upper barcode labels the icluster result, showing the distribution of samples according to inferred cluster. Lower barcode shows the intrinsic subtype (based on PAM50 classifier) of each sample. P-value is from a Fisher's exact test, comparing the distribution of the two luminal subtypes across the two clusters. Heatmap shows the *expression value* of these samples over the 4311 genes. Red indicates overexpression, whereas green indicates

underexpression.

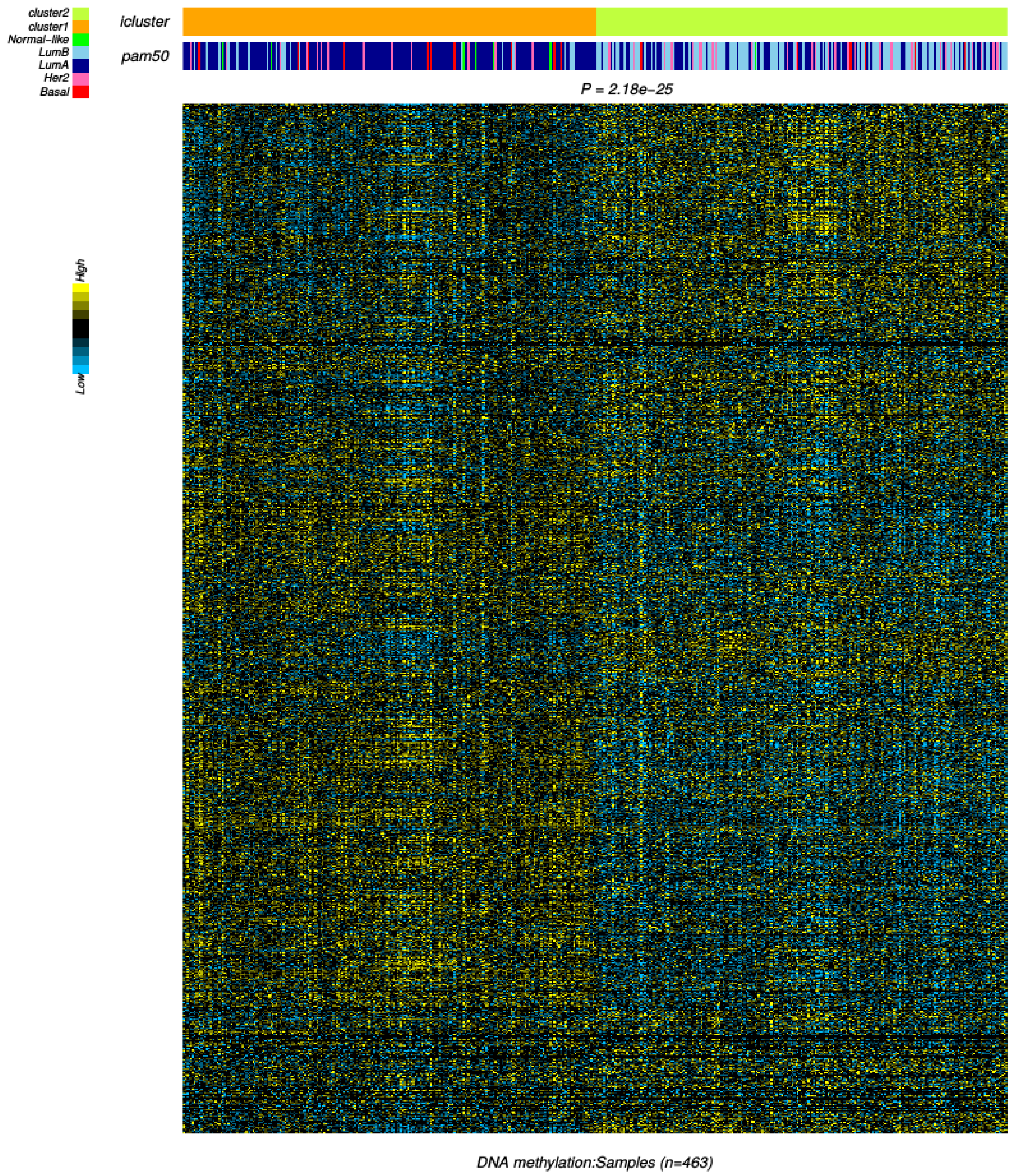

Fig.S3-partB: Integrative clustering of matched DNAm and mRNA data from 463 ER+ breast cancers using 4311 genes exhibiting an anti-correlative relation between differential methylation and differential expression (between normal and ER+ breast cancer). Upper barcode labels the icluster result, showing the distribution of samples according to inferred cluster. Lower barcode shows the intrinsic subtype (based on PAM50 classifier) of each sample. P-value is from a Fisher's exact test, comparing the distribution of the two luminal subtypes across

the two clusters. Heatmap shows the <u>*DNA methylation values*</u> of these samples over the 4311 genes. Yellow indicates hypermethylation, blue indicates hypomethylation.

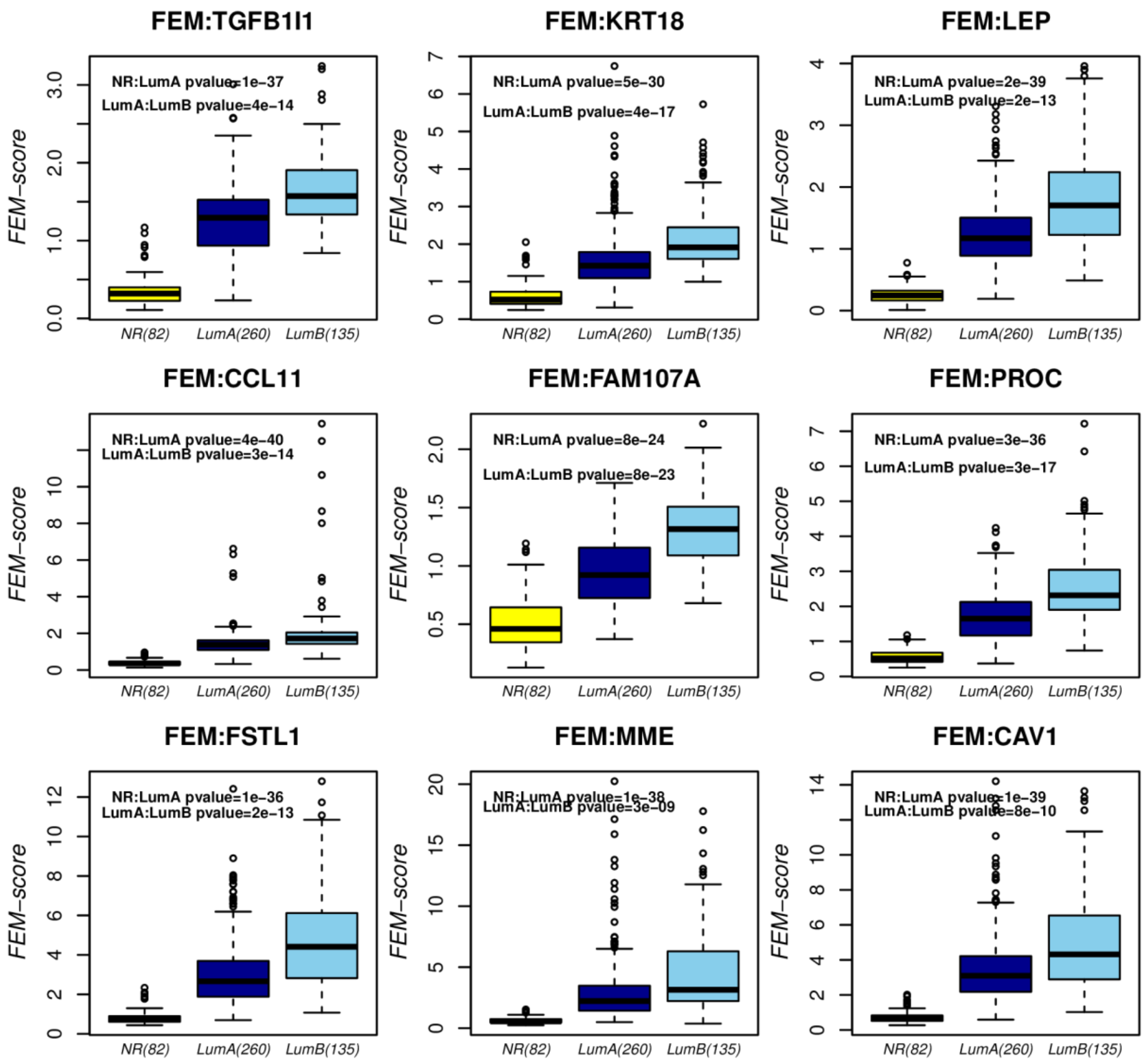

Fig.S4: Boxplots comparing the FEM scores of each FEM-module across the normal tissue samples (NR), luminal-A (lumA) and luminal-B (lumB) ER positive breast cancer from the TCGA breast cancer study. Number of samples in each group are indicated below boxplots. P-values are from a one-tailed Wilcoxon rank sum test.

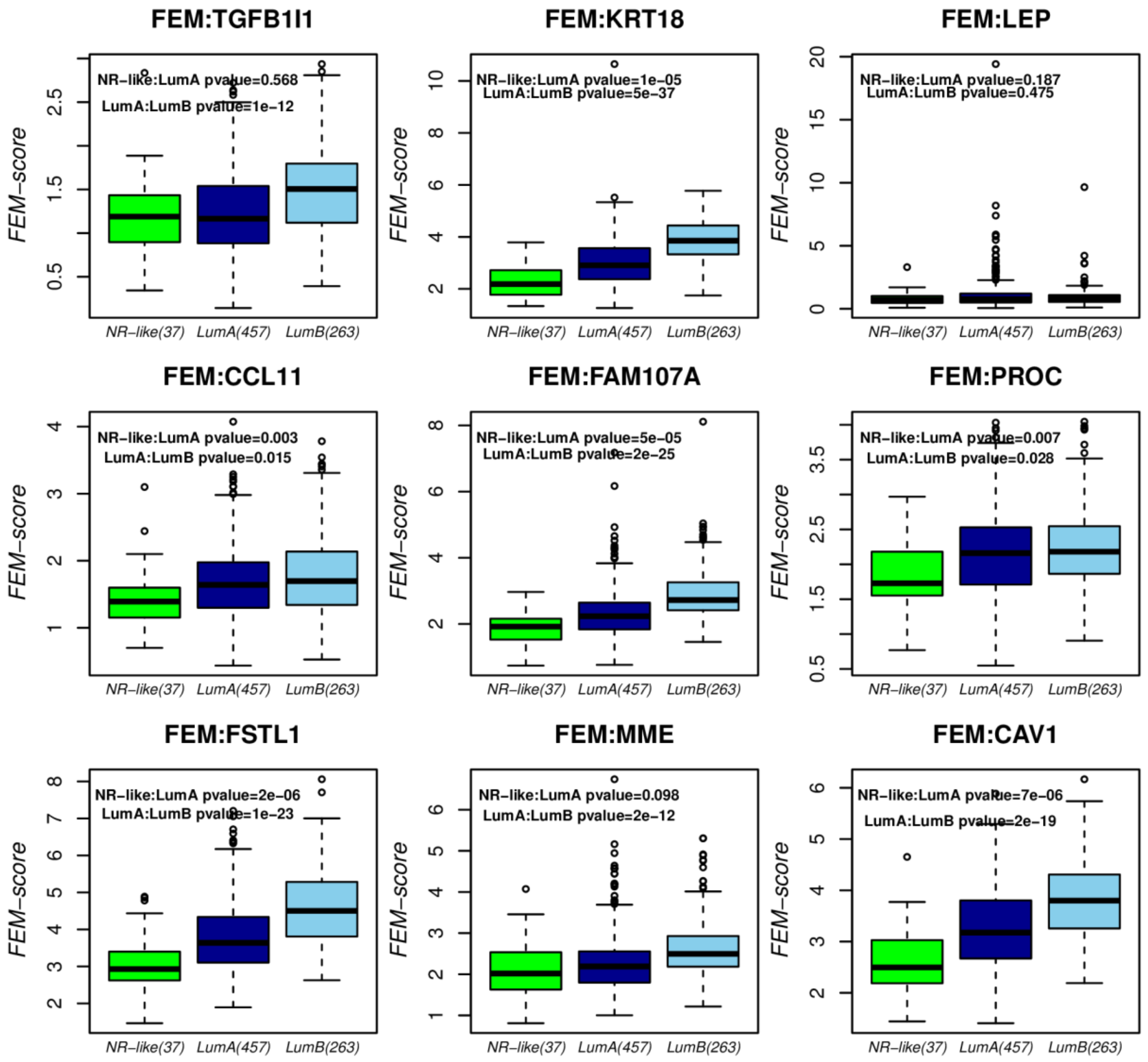

Fig.S5: FEM-scores of the FEM-modules inferred from the TCGA data in the METABRIC set. Boxplots compare the FEM scores across normal-like breast cancer (calibrated from normal-like subtype of TCGA dataset), luminal-A and luminal-B ER positive breast cancer. Because the METABRIC set only has mRNA expression data, the FEM scores were computed using only mRNA.Number of samples in each group are indicated below boxplots. P values are from a one-tailed Wilcoxon rank sum test.

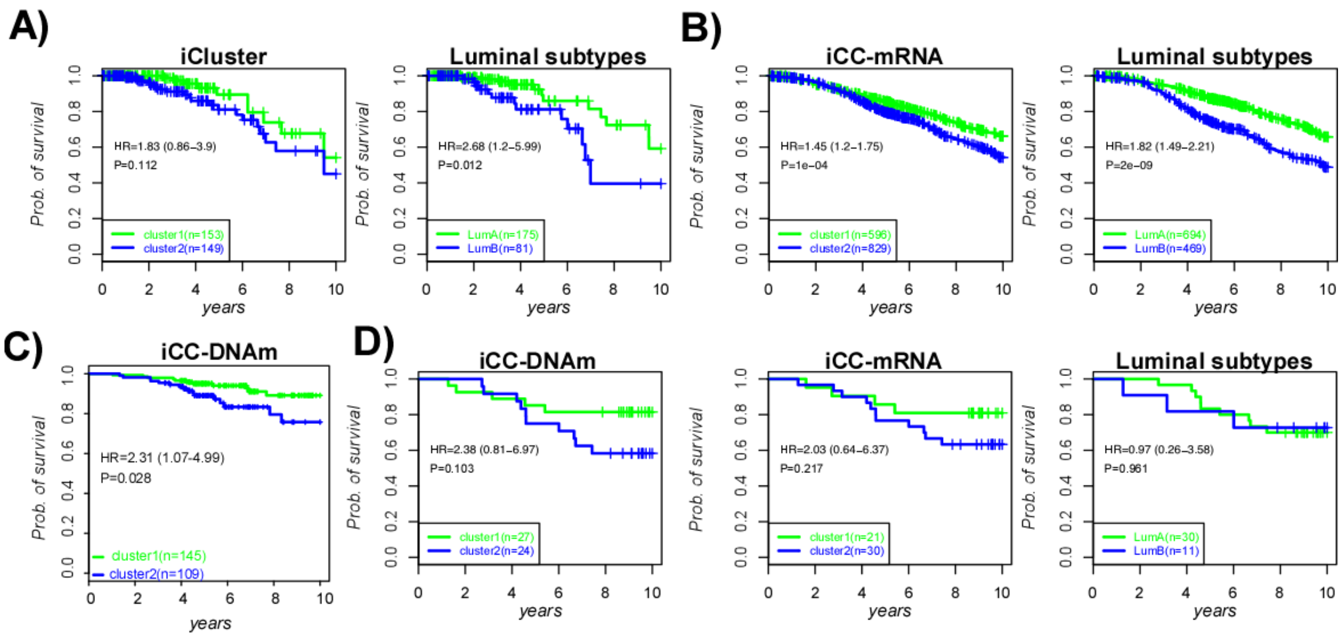

Fig.S6: **A)** Kaplan Meier Survival curves of the two integrative clusters predicted by iCluster on the matched TCGA Illumina 450k and RNA-Seq data, as well as those of luminal-A and B breast cancers. **B)** As A) but now for the predicted classification using the integrative cluster centroids (iCC) in the METABRIC expression set. **C)** As B) but now for the predicted classification in the “Germany” Illumina 450k DNAm set (this cohort had no expression data, so no luminal-classification is available). **D)** As B) but now for the predicted classification in the Fleischer data set for which there is both Illumina 450k DNAm and gene expression data (hence two different classifications iCC-DNAm and iCC-mRNA). In all panels, we provide the Hazard Ratio (HR), 95%CI and score-test P-values.

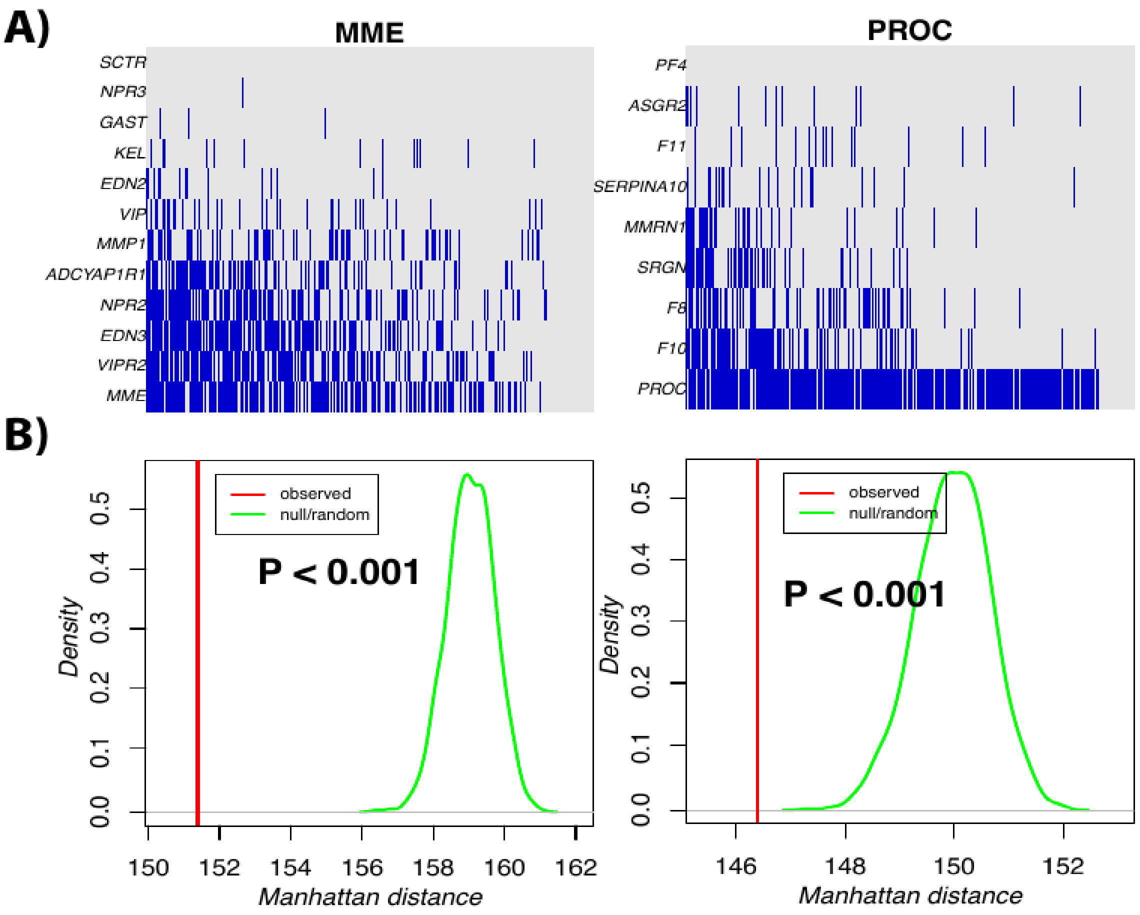
A)
MME
SCTR
NPR3
GAST
KEL
EDN2
VIP
MMP1
ADCYAP1R1
NPR2
EDN3
VIPR2
MME
PROC
PF4
ASGR2
F11
SERPINA10
MMRN1
SRGN
F8
F10
PROC
B)
observed
null/random
P < 0.001
Density
0.0
0.1
0.2
0.3
0.4
0.5
150
152
154
156
158
160
162
Manhattan distance
observed
null/random
P < 0.001
Density
146
148
150
152
Manhattan distance

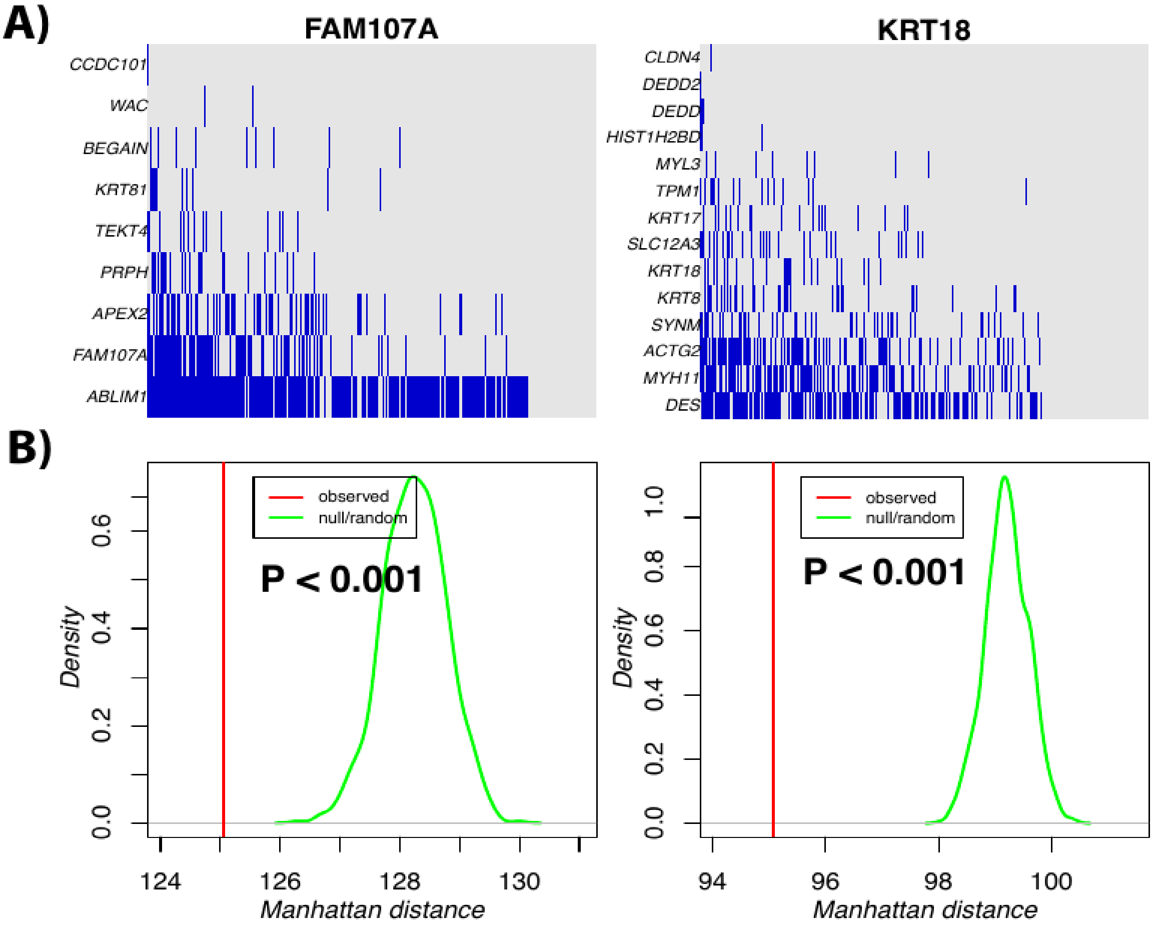
A)
FAM107A
CCDC101
WAC
BEGAIN
KRT81
TEKT4
PRPH
APEX2
FAM107A
ABLIM1
KRT18
CLDN4
DEDD2
DEDD
HIST1H2BD
MYL3
TPM1
KRT17
SLC12A3
KRT18
KRT8
SYNM
ACTG2
MYH11
DES
B)
observed
null/random
P < 0.001
Density
0.0
0.2
0.4
0.6
124
126
128
130
Manhattan distance
observed
null/random
P < 0.001
Density
0.0
0.2
0.4
0.6
0.8
1.0
94
96
98
100
Manhattan distance

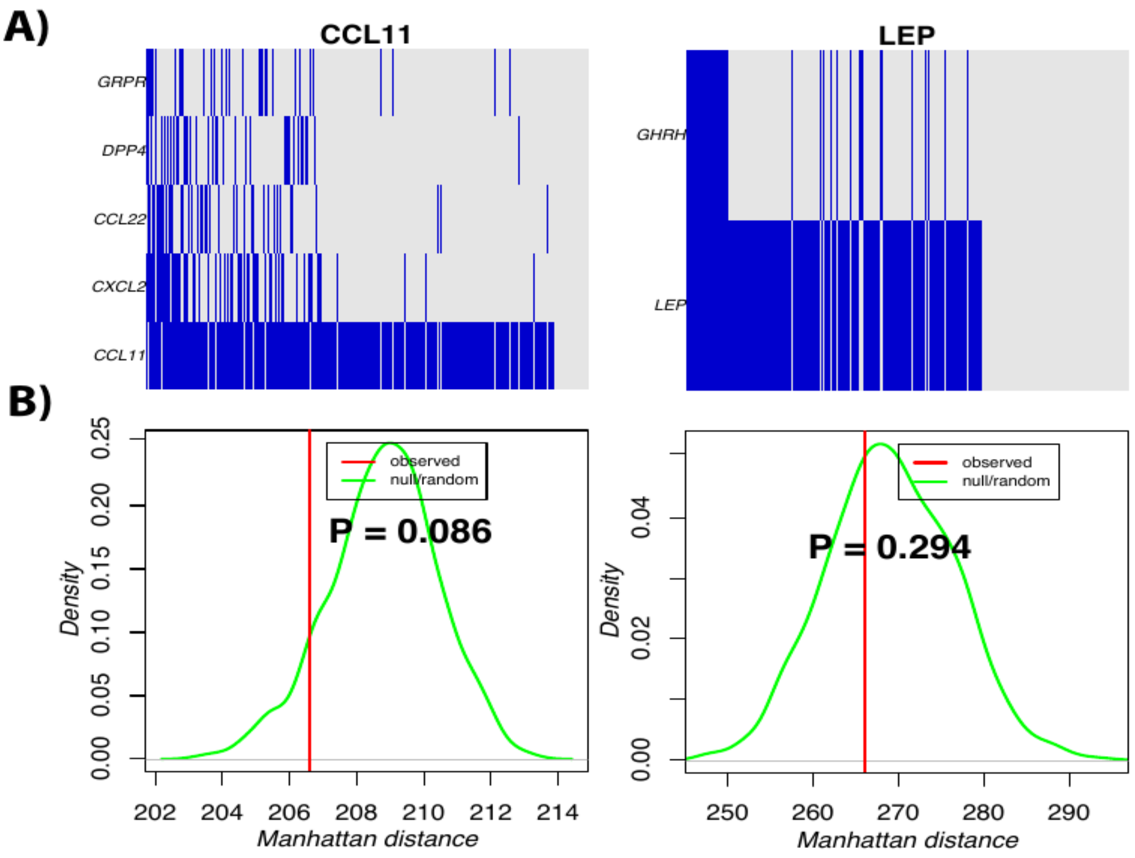

A)
CCL11
GRPR
DPP4
CCL22
CXCL2
CCL11
LEP
GHRH
LEP
B)
observed
null/random
P = 0.086
Density
0.00
0.05
0.10
0.15
0.20
0.25
202
204
206
208
210
212
214
Manhattan distance
observed
null/random
P = 0.294
Density
0.00
0.02
0.04
250
260
270
280
290
Manhattan distance

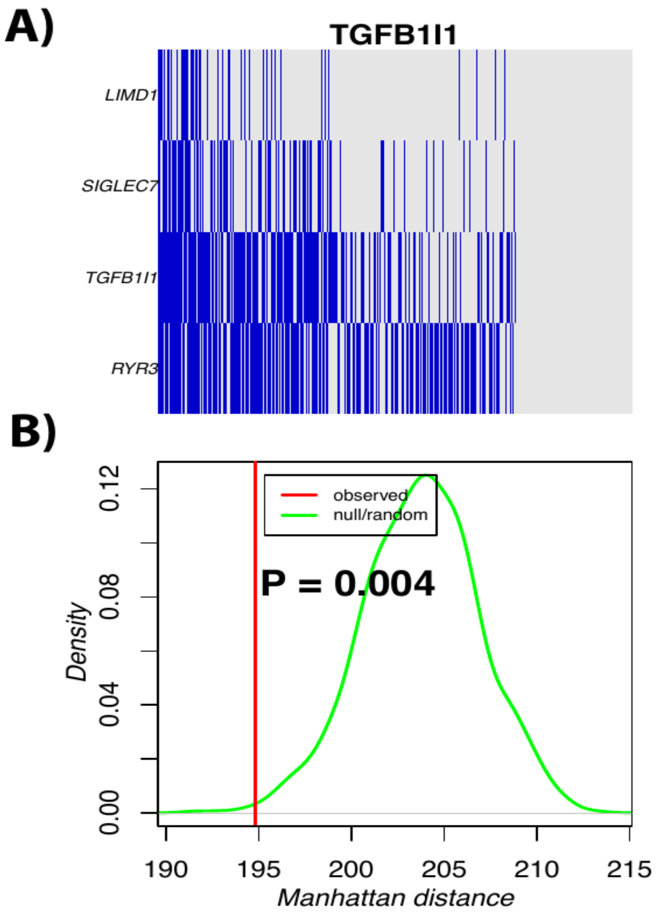

A)
TGFB1I1
LIMD1
SIGLEC7
TGFB1I1
RYR3
B)
observed
null/random
P = 0.004
Density
0.00
0.04
0.08
0.12
190
195
200
205
210
215
Manhattan distance

Fig.S7: A) Panels for each FEM depict heatmap representations of the binary deviation matrices with rows labeling FEM genes undergoing significant differential DNA methylation and differential expression, and with columns labeling tumour samples. In the heatmaps, a blue line indicates that the corresponding FEM gene in a given tumour, exhibits a significant deviation in DNAm and mRNA expression from the normal tissue reference. Grey indicates no significant deviation. **B)** The density plots for each FEM compare the observed average Manhattan distance of all gene pairs within a FEM (red line, as computed from the binary matrix representation shown in A)) with the corresponding Manhattan distance obtained by randomly permuting the samples within the binary matrix (green null curve depicts the density over 1000 different permutations). Estimate P-value is the fraction of permutations with an average Manhattan distance smaller than the observed distance.

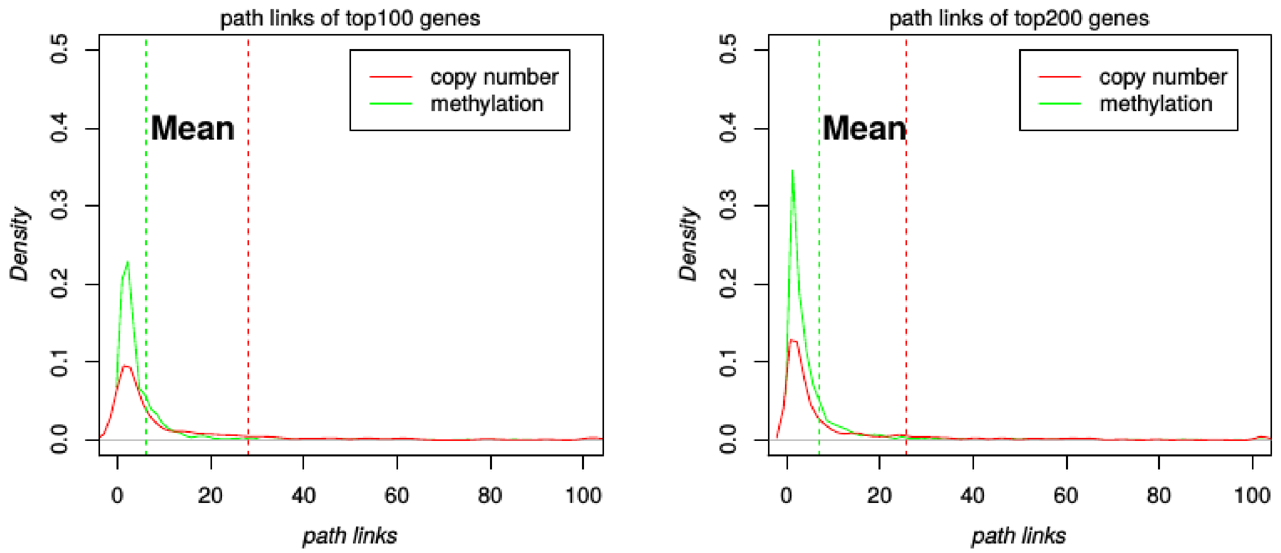

Fig.S8: Density plots of the shortest path, within a PPI network, between all gene pairs selected from the top 100 or top 200 differentially methylated or differentially amplified/deleted genes. The mean value of the shortest path between gene pairs is shown as a vertical line, with red indicating copy number and green indicating DNA methylation. Copy number and DNA methylation data are from TCGA breast cancer set.

SUPPLEMENTARY TABLES:

EntrezID(Seed)	Symbol(Seed)	Size	Mod	P	Genes
7041	**TGFB1I1**	13	5.0	0.018	TGFB1I1 TRAF4 GIT2 FKBP1A SVIL HIPK3 SIGLEC7 RYR3 LIMD1 RPP14 POP5 RPP25 TBC1D8
3875	**KRT18**	47	4.4	0.001	KRT18 H2AFX EXOC8 KRT17 TGM2 KRT8 ANP32B ACTG2 PIPOX MYLK2 RPS6KA3 HIST1H1C HIST1H2AG ACTN2 MYH7 PKD1 MYL3 DES CEP250 MYH13 MYH2 CACNA1S H1F0 HIST1H2BD MYH11 TGIF2LY TPM1 MYH3 HIST1H1D HIST1H1T DEDD TRO RHPN2 DNAJB6 TUT1 HMGCS2 WNK4 SYNM TRIM7 SYNC NEB KRT7 CLDN4 SLC12A3 DEDD2 CLDN2 ALG9
3952	**LEP**	13	4.6	0.049	LEP GHRL CRP UCN SIGLEC6 FCN2 GP6 GHRHR GHRH MLNR MLN CRHBP GHSR
6356	**CCL11**	12	5.1	0.018	CCL11 CCR2 ACKR2 DPP4 CCL22 CXCL2 CCR4 CCL28 GRP GRPR CCL25 CCR9
11170	**FAM107A**	16	4.6	0.036	FAM107A LDOC1 KRT15 TRIM37 BEGAIN HOOK2 ABLIM1 KRT81 USP2 WAC TEKT4 CCDC101 APEX2 ZNF417 PRPH ZNF253
5624	**PROC**	18	7.1	0	PROC F8 F5 SERPINA5 PROS1 THBD PROCR PF4 F10 SERPINA10 ASGR2 GAD2 MMRN1 F9 CERS2 F3 SRGN F11
11167	**FSTL1**	56	4.0	0.033	FSTL1 BMP4 BMP2 BMPR1A FST GREM2 BMPER MGP SOSTDC1 BMP6 BMP7 BAMBI CHRDL1 NOG GREM1 CHRD BMPR2 CCDC89 GDF5 TLL1 GDF6 INHBE ARSA TWSG1 LMX1A BMPR1B GDF9 BMP15 ADPGK ACVR2A ENG TGFB2 TGFBR3 DCAF12 CER1 OC90 MERTK FMOD TGFB3 TGFB1 BMP3 DPT HTRA1 GDF2 ACVRL1 TGFBRAP1 MOS SERPINB9 POMK NBEA LRP10 LSM10 SLBP LSM11 LTBP4 LSP1
25928	SOSTDC1	10	5.7	0.012	SOSTDC1 BMP4 BMP6 BAMBI BMP7 BMPER CHRDL1 FST INHBE GREM2

4311	**MME**	26	5.0	0.003	MME EDN1 NPPA GAST VIP EDN2 ADM NPPC ADCYAP1 VIPR2 ADCYAP1R1 NPR2 KEL MMP1 EDNRA EDN3 CMA1 EDNRB NPR3 CYSLTR2 SCTR SCT VIPR1 GNA12 ECE1 NPPB
857	**CAV1**	58	3.7	0.046	CAV1 EDNRB GRK5 TNFRSF1B TNFRSF1A WNT3A LRP6 FZD5 F2R CAV2 GJA1 GP2 GJA3 MALL BST1 PROCR GJB2 HTR1F WIF1 SLC1A3 KCTD12 ANTXR2 LTA WNT1 FZD1 FZD9 WNT7A LTB SFRP1 FZD6 WNT4 WNT2 RSPO1 DKK1 DKK2 RYK IGFBP4 SFRP2 FZD8 ATP6AP2 WNT3 WNT5A PI4K2A LTBR WNT7B GJB6 ANTXR1 GJA5 FZD2 LRP5 FZD10 MESDC2 AVPR1B AVPR1A AVP TNFRSF25 TNFSF15 PIP4K2B
1674	DES	47	4.4	0.004	DES H2AFX KRT18 KRT17 KRT8 MYLK2 HIST1H1C HIST1H2AG ACTN2 MYH7 PKD1 MYL3 CEP250 MYH13 MYH2 CACNA1S H1F0 HIST1H2BD MYH11 TGIF2LY TPM1 MYH3 HIST1H1D HIST1H1T NEB SYNM SYNC TRIM7 TUT1 HMGCS2 TRO WNK4 DEDD DNAJB6 PIPOX RHPN2 ANP32B KRT7 ACTG2 TGM2 EXOC8 CLDN4 SLC12A3 RPS6KA3 DEDD2 CLDN2 ALG9

Table.S1: Properties of the 11 ER+ breast cancer associated FEMs, of which 9 are non-redundant (shown in boldface). For each FEM, we provide the Entrez gene ID and gene symbol of the seed gene, the size of the FEM, its modularity score, the P-value of significance of the modularity score (as assessed using 1000 Monte-Carlo randomisations), and the gene symbols of all genes making up the FEM.

TGFB1I1 (13 genes)

EntrezID	Symbol	stat(DNAm)	P(DNAm)	stat(mRNA)	P(mRNA)	stat(Int)
7041	TGFB1I1	29.42	0	-10.55	2.00E-24	17.81
9618	TRAF4	0.95	0.344	12.63	1.00E-33	0
9815	GIT2	-1.61	0.108	-8.54	6.00E-17	0
2280	FKBP1A	0.68	0.494	0.2	0.844	0
6840	SVIL	-0.18	0.854	-14.9	1.00E-44	0
10114	HIPK3	-0.33	0.745	-8.01	4.00E-15	0
27036	SIGLEC7	-16.67	5.00E-51	13.82	3.00E-39	12.4
6263	RYR3	8.49	2.00E-16	-32.38	0	13.76
8994	LIMD1	-6.46	2.00E-10	6.45	2.00E-10	5.13
11102	RPP14	-0.92	0.357	-4.35	2.00E-05	0
51367	POP5	-2.19	0.029	0.38	0.701	1.21
54913	RPP25	3.74	2.00E-04	5.92	5.00E-09	0

11138	TBC1D8	-0.65	0.515	0.05	0.958	0.34

KRT18 (47 genes)

EntrezID	Symbol	stat(DNAm)	P(DNAm)	stat(mRNA)	P(mRNA)	stat(Int)
3875	KRT18	-23.9	8.00E-88	19.46	8.00E-70	17.67
3014	H2AFX	4.26	2.00E-05	19.62	8.00E-71	0
149371	EXOC8	-0.35	0.725	2.29	0.022	0.85
3872	KRT17	5.9	6.00E-09	-10.25	3.00E-23	5.96
7052	TGM2	0.67	0.505	4.72	3.00E-06	0
3856	KRT8	-12.57	4.00E-32	18.8	5.00E-66	11.81
10541	ANP32B	-1.02	0.307	0.78	0.433	0.74
72	ACTG2	6.39	3.00E-10	-19.95	1.00E-72	9.06
51268	PIPOX	4.04	6.00E-05	7.4	3.00E-13	0
85366	MYLK2	9.35	2.00E-19	11.98	1.00E-30	0
6197	RPS6KA3	-2.93	0.003	-16.47	6.00E-53	0
3006	HIST1H1C	-1.13	0.258	12.06	5.00E-31	4.11
8969	HIST1H2AG	-1.09	0.275	10.24	3.00E-23	3.56
88	ACTN2	13.99	2.00E-38	3.81	1.00E-04	0
4625	MYH7	-3.29	0.001	-1.3	0.193	0
5310	PKD1	-2.38	0.018	-7.67	5.00E-14	0
4634	MYL3	7.31	9.00E-13	-7.31	6.00E-13	5.8
1674	DES	15.98	1.00E-47	-14.37	5.00E-42	12.21
11190	CEP250	0.45	0.656	10.79	2.00E-25	0
8735	MYH13	-14.16	4.00E-39	0.88	0.381	7.34
4620	MYH2	-7.55	2.00E-13	-9.12	6.00E-19	0
779	CACNA1S	-1.64	0.102	-1.39	0.164	0
3005	H1F0	5.55	4.00E-08	9.8	1.00E-21	0
3017	HIST1H2BD	-2.91	0.004	20.11	1.00E-73	7.37
4629	MYH11	8.31	7.00E-16	-21.65	8.00E-83	10.52
90655	TGIF2LY	-15.47	3.00E-45	0.7	0.487	7.94
7168	TPM1	4.69	3.00E-06	-6.72	3.00E-11	4.32
4621	MYH3	-4.18	3.00E-05	-12.46	8.00E-33	0
3007	HIST1H1D	2.49	0.013	10.27	2.00E-23	0
3010	HIST1H1T	0.73	0.467	4.01	7.00E-05	0
9191	DEDD	-4.51	8.00E-06	7.39	4.00E-13	4.43
7216	TRO	-8.1	3.00E-15	-10.07	1.00E-22	0
85415	RHPN2	-0.19	0.853	3.62	3.00E-04	1.16
10049	DNAJB6	-4.35	2.00E-05	0.91	0.363	2.44
64852	TUT1	-1.6	0.11	3.08	0.002	1.71
3158	HMGCS2	-10.45	2.00E-23	-5.75	1.00E-08	0
65266	WNK4	2.01	0.045	5.21	2.00E-07	0
23336	SYNM	3.69	2.00E-04	-27.32	0	9.88
81786	TRIM7	1.66	0.097	-2.3	0.022	1.51
81493	SYNC	1.08	0.28	0.75	0.456	0
4703	NEB	-3.26	0.001	0	0.999	0
3855	KRT7	5.52	5.00E-08	-1.41	0.159	3.17
1364	CLDN4	-16.29	3.00E-49	8.4	2.00E-16	10.61
6559	SLC12A3	-2.58	0.01	4.92	1.00E-06	2.74
162989	DEDD2	-2.62	0.009	12.74	4.00E-34	5.05
9075	CLDN2	-1.83	0.068	-10.99	2.00E-26	0
79796	ALG9	-1.65	0.1	-8.64	3.00E-17	0

LEP (13 genes)

EntrezID	Symbol	stat(DNAm)	P(DNAm)	stat(mRNA)	P(mRNA)	stat(Int)
3952	LEP	22.22	4.00E-79	-21.8	9.00E-84	17.52
51738	GHRL	1.5	0.135	3.61	3.00E-04	0
1401	CRP	-14.98	6.00E-43	0.45	0.655	7.62
7349	UCN	15.61	6.00E-46	11.69	2.00E-29	0
946	SIGLEC6	3.62	3.00E-04	1.32	0.188	0
2220	FCN2	-0.17	0.862	-6.15	1.00E-09	0
51206	GP6	5.05	6.00E-07	2.7	0.007	0
2692	GHRHR	-13.5	3.00E-36	1.17	0.244	7.09
2691	GHRH	-5.63	3.00E-08	4.65	4.00E-06	4.18
2862	MLNR	9.75	8.00E-21	3.36	8.00E-04	0
4295	MLN	-6.95	1.00E-11	-0.79	0.43	0
1393	CRHBP	-5.38	1.00E-07	-20.21	3.00E-74	0
2693	GHSR	14.82	3.00E-42	0.01	0.989	0

CCL11 (12 genes)

EntrezID	Symbol	stat(DNAm)	P(DNAm)	stat(mRNA)	P(mRNA)	stat(Int)
6356	CCL11	-19.8	1.00E-66	24.64	0	17.14
729230	CCR2	-3.1	0.002	-1.68	0.092	0
1238	ACKR2	-1.76	0.079	-9.1	7.00E-19	0
1803	DPP4	5.94	5.00E-09	-12.98	3.00E-35	6.78
6367	CCL22	-11.61	4.00E-28	3.6	3.00E-04	6.86
2920	CXCL2	2.19	0.029	-23.89	1.00E-96	8.12
1233	CCR4	11.92	2.00E-29	11.68	3.00E-29	0
56477	CCL28	0.05	0.956	-15.93	5.00E-50	4.71
2922	GRP	11.99	1.00E-29	4.56	6.00E-06	0
2925	GRPR	-2.98	0.003	2.96	0.003	2.36
6370	CCL25	-1.77	0.077	3.54	4.00E-04	1.93
10803	CCR9	1.84	0.066	-1.69	0.091	1.42

FAM107A (16 genes)

EntrezID	Symbol	stat(DNAm)	P(DNAm)	stat(mRNA)	P(mRNA)	stat(Int)
11170	FAM107A	16.42	7.00E-50	-26.92	0	16.13
23641	LDOC1	3.76	2.00E-04	2.38	0.017	0
3866	KRT15	-0.59	0.556	-9.41	5.00E-20	0
4591	TRIM37	0.77	0.443	8.3	4.00E-16	0
57596	BEGAIN	4.37	1.00E-05	-6.71	3.00E-11	4.16
29911	HOOK2	-0.67	0.503	-4.11	4.00E-05	0
3983	ABLIM1	16.79	1.00E-51	-20.46	1.00E-75	14.41
3887	KRT81	19.18	2.00E-63	-4.11	4.00E-05	10.8
9099	USP2	0.88	0.378	-9.69	4.00E-21	3.29
51322	WAC	9.17	9.00E-19	-6.31	5.00E-10	6.44
150483	TEKT4	-6.02	3.00E-09	5	7.00E-07	4.48
112869	CCDC101	-3.23	0.001	4.68	3.00E-06	2.99
27301	APEX2	-5.76	1.00E-08	16.41	1.00E-52	7.71
147687	ZNF417	0.97	0.334	-0.15	0.881	0.53
5630	PRPH	4.92	1.00E-06	-5.44	7.00E-08	4.06
56242	ZNF253	0.45	0.651	2.59	0.01	0

PROC (18 genes)

EntrezID	Symbol	stat(DNAm)	P(DNAm)	stat(mRNA)	P(mRNA)	stat(Int)
5624	PROC	-16.38	1.00E-49	24.52	########	15.4
2157	F8	7.17	2.00E-12	-20.94	1.00E-78	9.74

2153	F5	6.92	1.00E-11	6.54	1.00E-10	0
5104	SERPINA5	-8.11	3.00E-15	0.34	0.735	4.15
5627	PROS1	1.58	0.115	-23.53	3.00E-94	7.71
7056	THBD	1.52	0.129	-10.1	1.00E-22	3.73
10544	PROCR	0.36	0.717	-9.06	9.00E-19	2.85
5196	PF4	2.21	0.028	-4.8	2.00E-06	2.51
2159	F10	12.17	2.00E-30	-19.55	2.00E-70	11.83
51156	SERPINA10	-2.13	0.033	2.23	0.026	1.72
433	ASGR2	-6.07	2.00E-09	3.8	2.00E-04	4.16
2572	GAD2	7.29	1.00E-12	-0.4	0.689	3.77
22915	MMRN1	3.85	1.00E-04	-18.12	4.00E-62	7.26
2158	F9	1.34	0.181	1.28	0.201	0
29956	CERS2	3.5	5.00E-04	21.16	7.00E-80	0
2152	F3	-1.02	0.307	-16.42	1.00E-52	0
5552	SRGN	8.75	2.00E-17	-6.1	2.00E-09	6.17
2160	F11	-5.42	9.00E-08	2.67	0.008	3.49

FSTL1 (56 genes)

EntrezID	Symbol	stat(DNAm)	P(DNAm)	stat(mRNA)	P(mRNA)	stat(Int)
11167	FSTL1	22.44	3.00E-80	-11.54	1.00E-28	14.61
652	BMP4	-0.09	0.929	-4.93	1.00E-06	0
650	BMP2	7.47	3.00E-13	-21.19	4.00E-80	9.96
657	BMPR1A	1.58	0.114	-10.16	6.00E-23	3.78
10468	FST	7.72	5.00E-14	-4.44	1.00E-05	5.16
64388	GREM2	3.51	5.00E-04	-13.72	9.00E-39	5.79
168667	BMPER	6.99	8.00E-12	-16.04	1.00E-50	8.21
4256	MGP	3.61	3.00E-04	-8.82	7.00E-18	4.4
25928	SOSTDC1	19.56	2.00E-65	-16.29	5.00E-52	14.57
654	BMP6	5.46	7.00E-08	-20.74	2.00E-77	8.83
655	BMP7	6.83	2.00E-11	-3.48	5.00E-04	4.44
25805	BAMBI	0.31	0.753	3.04	0.002	0
91851	CHRDL1	9.74	8.00E-21	-20.88	3.00E-78	11.01
9241	NOG	-0.54	0.586	-3.45	6.00E-04	0
26585	GREM1	12.28	7.00E-31	6.47	2.00E-10	0
8646	CHRD	-3.96	8.00E-05	7.46	2.00E-13	4.17
659	BMPR2	2.59	0.01	-7.33	5.00E-13	3.45
220388	CCDC89	12.86	2.00E-33	-5.74	1.00E-08	8.12
8200	GDF5	2	0.046	-14.05	2.00E-40	5.13
7092	TLL1	1.91	0.057	-11.14	5.00E-27	4.23
392255	GDF6	6.37	4.00E-10	0.77	0.443	0
83729	INHBE	-0.66	0.51	2.44	0.015	1.05
410	ARSA	-0.07	0.941	3.91	1.00E-04	1.19
57045	TWSG1	6.31	6.00E-10	2.33	0.02	0
4009	LMX1A	10.37	3.00E-23	-13.33	7.00E-37	9.11
658	BMPR1B	3.44	6.00E-04	9.26	2.00E-19	0
2661	GDF9	-1.67	0.095	7.03	4.00E-12	2.9
9210	BMP15	-4.7	3.00E-06	0.5	0.62	2.5
83440	ADPGK	-3.74	2.00E-04	6.66	5.00E-11	3.83
92	ACVR2A	-3.11	0.002	-16.04	1.00E-50	0
2022	ENG	13.74	3.00E-37	-7.06	3.00E-12	8.94
7042	TGFB2	0.81	0.418	-4.63	4.00E-06	1.77
7049	TGFBR3	-0.2	0.845	-19.23	2.00E-68	0
25853	DCAF12	-3.81	2.00E-04	6.06	2.00E-09	3.69

9350	CER1	-5.92	6.00E-09	-0.89	0.375	0
729330	OC90	-12.16	2.00E-30	-1.19	0.234	0
10461	MERTK	2.7	0.007	-12.87	1.00E-34	5.14
2331	FMOD	2.07	0.039	-10.39	7.00E-24	4.09
7043	TGFB3	3.52	5.00E-04	10.51	2.00E-24	0
7040	TGFB1	1.79	0.074	12.1	3.00E-31	0
651	BMP3	6.46	2.00E-10	-18.71	2.00E-65	8.73
1805	DPT	12.06	6.00E-30	-18.19	2.00E-62	11.38
5654	HTRA1	1.19	0.236	-1.47	0.143	1.02
2658	GDF2	-5.21	3.00E-07	1.03	0.305	2.91
94	ACVRL1	8.35	5.00E-16	-13.34	6.00E-37	8.1
9392	TGFBRAP1	0.98	0.326	-1.83	0.068	1.03
4342	MOS	10.47	1.00E-23	-1.28	0.201	5.61
5272	SERPINB9	0.4	0.688	-4.5	8.00E-06	1.52
84197	POMK	-0.63	0.532	4.02	6.00E-05	1.49
26960	NBEA	5.2	3.00E-07	-2.84	0.005	3.44
26020	LRP10	-2.28	0.023	-0.51	0.611	0
84967	LSM10	-0.23	0.817	8.51	8.00E-17	2.62
7884	SLBP	3.2	0.001	9.58	1.00E-20	0
134353	LSM11	-2.1	0.036	2.71	0.007	1.85
8425	LTBP4	-7.85	2.00E-14	-19.94	1.00E-72	0
4046	LSP1	-9.81	4.00E-21	-1.32	0.186	0

MME (26 genes)

EntrezID	Symbol	stat(DNAm)	P(DNAm)	stat(mRNA)	P(mRNA)	stat(Int)
4311	MME	9.87	3.00E-21	-26.63	0	12.77
1906	EDN1	0.35	0.724	-15.58	3.00E-48	4.76
4878	NPPA	2.56	0.011	0.42	0.677	0
2520	GAST	-4.58	6.00E-06	4.03	6.00E-05	3.47
7432	VIP	3.32	0.001	-14.49	1.00E-42	5.92
1907	EDN2	-7.33	8.00E-13	13.98	5.00E-40	7.77
133	ADM	0.38	0.701	-15.17	5.00E-46	4.65
4880	NPPC	2.33	0.02	2.72	0.007	0
116	ADCYAP1	13.49	4.00E-36	-1.14	0.254	7.08
7434	VIPR2	11.54	9.00E-28	-3.85	1.00E-04	6.9
117	ADCYAP1R1	5.33	1.00E-07	-20.16	6.00E-74	8.59
4882	NPR2	16.25	5.00E-49	-20.47	8.00E-76	14.15
3792	KEL	-3.91	1.00E-04	2.21	0.027	2.6
4312	MMP1	-6.56	1.00E-10	18.82	4.00E-66	8.81
1909	EDNRA	14.62	3.00E-41	1.59	0.111	0
1908	EDN3	14.62	3.00E-41	-16.71	3.00E-54	12.22
1215	CMA1	-8.52	1.00E-16	-15.49	9.00E-48	0
1910	EDNRB	-13.04	4.00E-34	-22.32	6.00E-87	0
4883	NPR3	2.79	0.005	-9.38	6.00E-20	4.15
57105	CYSLTR2	-6.46	2.00E-10	-1.62	0.106	0
6344	SCTR	12.04	7.00E-30	-12.63	1.00E-33	9.73
6343	SCT	18.14	3.00E-58	14.4	4.00E-42	0
7433	VIPR1	-3.35	9.00E-04	-10.51	2.00E-24	0
2768	GNA12	-1.3	0.195	-3.22	0.001	0
1889	ECE1	-3.28	0.001	-6.18	1.00E-09	0
4879	NPPB	2.73	0.007	1.71	0.088	0

CAV1 (58 genes)

EntrezID	Symbol	stat(DNAm)	P(DNAm)	stat(mRNA)	P(mRNA)	stat(Int)
857	CAV1	7.58	1.00E-13	-29.98	0	12.6
1910	EDNRB	-13.04	4.00E-34	-22.32	6.00E-87	0
2869	GRK5	-1.13	0.259	-18.28	5.00E-63	0
7133	TNFRSF1B	5.92	6.00E-09	-9.97	4.00E-22	5.89
7132	TNFRSF1A	-3.55	4.00E-04	-5	7.00E-07	0
89780	WNT3A	7.91	1.00E-14	0.87	0.384	0
4040	LRP6	0.51	0.61	-12.91	7.00E-35	4.05
7855	FZD5	0.35	0.727	-12.69	7.00E-34	3.91
2149	F2R	1.71	0.088	9.56	1.00E-20	0
858	CAV2	0.41	0.683	-30.6	0	9.2
2697	GJA1	4.91	1.00E-06	0.57	0.571	0
2813	GP2	-19.24	8.00E-64	8.96	2.00E-18	12.25
2700	GJA3	3.79	2.00E-04	10.61	9.00E-25	0
7851	MALL	14.75	7.00E-42	-14.05	2.00E-40	11.51
683	BST1	7.08	4.00E-12	-10.87	8.00E-26	6.74
10544	PROCR	0.36	0.717	-9.06	9.00E-19	2.85
2706	GJB2	2.8	0.005	22.46	9.00E-88	0
3355	HTR1F	-2.3	0.022	-2.55	0.011	0
11197	WIF1	10.79	8.00E-25	-18.77	7.00E-66	10.91
6507	SLC1A3	13.61	1.00E-36	-11.24	2.00E-27	10.11
115207	KCTD12	1.75	0.08	-19.08	1.00E-67	6.49
118429	ANTXR2	4.57	6.00E-06	-19.52	3.00E-70	8.03
4049	LTA	-5.38	1.00E-07	9.16	4.00E-19	5.38
7471	WNT1	8.84	1.00E-17	-4.27	2.00E-05	5.68
8321	FZD1	-0.8	0.424	-6.92	9.00E-12	0
8326	FZD9	4.12	4.00E-05	-9.52	2.00E-20	4.86
7476	WNT7A	5.8	1.00E-08	1.97	0.05	0
4050	LTB	-2.02	0.044	8.64	3.00E-17	3.55
6422	SFRP1	12.35	3.00E-31	-21.51	5.00E-82	12.5
8323	FZD6	-1.66	0.098	-2.18	0.029	0
54361	WNT4	1.81	0.071	3	0.003	0
7472	WNT2	16.17	1.00E-48	7.22	1.00E-12	0
284654	RSPO1	6.41	3.00E-10	-9.21	3.00E-19	5.91
22943	DKK1	-0.29	0.769	-1.07	0.285	0
27123	DKK2	8.39	4.00E-16	-0.8	0.424	4.43
6259	RYK	0.87	0.386	-14.09	1.00E-40	4.58
3487	IGFBP4	1.69	0.092	-0.45	0.653	0.98
6423	SFRP2	19.57	2.00E-65	4.79	2.00E-06	0
8325	FZD8	2.87	0.004	-4.71	3.00E-06	2.82
10159	ATP6AP2	5.18	3.00E-07	8.04	3.00E-15	0
7473	WNT3	1.93	0.054	7.04	4.00E-12	0
7474	WNT5A	0.28	0.782	0.44	0.663	0
55361	PI4K2A	-0.92	0.359	1.53	0.127	0.91
4055	LTBR	3.85	1.00E-04	4.52	7.00E-06	0
7477	WNT7B	-1.19	0.236	13.06	1.00E-35	4.43
10804	GJB6	4.75	3.00E-06	-0.9	0.368	2.64
84168	ANTXR1	1.62	0.105	-1.01	0.315	1.11
2702	GJA5	4.79	2.00E-06	-4.04	6.00E-05	3.58
2535	FZD2	14.88	2.00E-42	16.78	1.00E-54	0
4041	LRP5	-2.53	0.012	-10.13	8.00E-23	0
11211	FZD10	7.63	1.00E-13	-0.64	0.523	4
23184	MESDC2	-2.68	0.008	8.08	2.00E-15	3.72

553	AVPR1B	-6.42	3.00E-10	0.2	0.842	3.27
552	AVPR1A	18.95	2.00E-62	-9.49	2.00E-20	12.26
551	AVP	-4.4	1.00E-05	4.38	1.00E-05	3.49
8718	TNFRSF25	1.4	0.163	-0.92	0.358	0.97
9966	TNFSF15	-7.95	1.00E-14	-0.76	0.448	0
8396	PIP4K2B	-0.55	0.582	-3.98	7.00E-05	0

Table.S2: Details of the 9 non-redundant ER+ breast cancer associated FEMs. For each FEM, labeled by its seed gene, we provide details of all genes making up the FEM, including their Entrez gene ID, their gene symbol, the t-statistic and P-value of differential DNA methylation and gene expression, as well as the integrated statistic.

Data Types	Clusters predicted	Adjusted Rand Index of luminal subtypes
DNAm + mRNA (TCGA)	2	0.262
Copy number+mRNA (METABRIC)	10	0.072
Copy number +mRNA IC1 and IC3 (METABRIC)	NA	0.507

Table.S3: Table of ARI (Adjusted Rand Index) values showing the classification accuracy of luminal subtypes across inferred clusters and using different data types. The number of clusters were predicted using the POD score of the icluster algorithm. Last row gives the ARI value for intrinsic clusters 1 and 3 of the METABRIC study (Curtis et al Nature 2012).

Normal – LumA subtype (FEM)

FEM Seed	Size	Mod(LumA)	P(LumA)	Mod(LumB)	P(LumB)
KRT18	49	8.38	0.009	4.29	0.003
TGFB1I1	13	9.71	0.025	5.16	0.012
CCL11	12	10.18	0.02	4.98	0.021
LEP	13	8.96	0.049	3.90	0.114
PROC	18	13.92	<0.001	6.89	0.001
FAM107A	16	9.18	0.034	4.7	0.049
SOSTDC1	10	10.99	0.011	5.95	0.006
FSTL1	56	7.60	0.041	4.03	0.02
MME	23	9.41	0.009	5.76	0.002
CAV1	58	7.46	0.049	3.69	0.044

Normal – LumB subtype (FEM)

FEM Seed	Size	Mod(LumB)	P(LumB)	Mod(LumA)	P(LumA)
TGFB1I1	13	10.32	0.02	4.854	0.028
FAM107A	19	8.98	0.031	4.407	0.035
CCL11	16	8.85	0.041	3.888	0.114

KRT18	49	8.51	0.007	4.138	0.005
SOSTDC1	57	8.01	0.024	3.778	0.053
FSTL1	57	8.01	0.03	3.778	0.039
LNX1	27	10.90	0.011	2.341	0.582
PROC	24	12.90	<0.001	6.548	<0.001
VWF	13	11.44	0.009	6.113	0.002
CAV1	60	7.59	0.036	3.846	0.021
MYH11	49	8.51	0.011	4.138	0.007
MME	27	10.87	<0.001	4.304	0.031
GPRASP1	36	10.63	0.007	4.613	0.022
DES	49	8.51	0.008	4.138	0.008

Table.S4: Cross-validation of luminal-subtype specific FEMs. Top table lists the FEMs inferred by comparing luminal-A ER+ breast cancers to normals, listing their modularities and Monte-Carlo estimated P-values of significance, plus their modularities and P-values obtained by comparing luminal-B ER+ breast cancer to normals. Similarly, the lower table lists the FEMs inferred by comparing luminal-A ER+ breast cancers to normals, listing their modularities and Monte-Carlo estimated P-values of significance, plus their modularities and P-values obtained by comparing luminal-A ER+ breast cancer to normals. Observe how most of the luminal-specific FEMs validate in the other luminal-subtype, indicating that both luminal subtypes share the same FEMs.
